# Liquid Biopsy Frontiers in Pancreatic Cancer: Insights from Circulating Cell-Free Nucleic Acids

**DOI:** 10.3390/cells15100904

**Published:** 2026-05-14

**Authors:** Maria Latiano, Maria De Angelis, Anna Latiano, Orazio Palmieri, Tiziana Pia Latiano, Marco Donatello Delcuratolo, Matteo Tardio, Francesca Bazzocchi, Marco Gentile, Fulvia Terracciano, Grazia Anna Niro, Francesca Tavano

**Affiliations:** 1 Gastrointestinal Disorders Research Unit, Fondazione “Casa Sollievo della Sofferenza” IRCCS Hospital, 71013 San Giovanni Rotondo, Italy; 2 Oncology Unit, Fondazione “Casa Sollievo della Sofferenza” IRCCS Hospital, 71013 San Giovanni Rotondo, Italy; 3 Abdominal Surgery Unit, Fondazione “Casa Sollievo della Sofferenza” IRCCS Hospital, 71013 San Giovanni Rotondo, Italy; 4 Division of Gastroenterology and Endoscopy, Fondazione “Casa Sollievo della Sofferenza” IRCCS Hospital, 71013 San Giovanni Rotondo, Italy

**Keywords:** pancreatic cancer, circulating cell-free DNA, circulating tumor DNA, circulating cell-free RNA, diagnosis, prognosis, monitoring

## Abstract

Pancreatic cancer (PC) remains one of the most aggressive and lethal malignancies worldwide, largely due to late diagnosis, aggressive biology, limited therapeutic options and responsiveness. Conventional diagnostic and monitoring strategies, including imaging and serum biomarkers such as CA 19-9, provide limited sensitivity for early detection and suboptimal accuracy for the dynamic assessment of treatment response and disease evolution. These limitations highlight the urgent need for innovative, minimally invasive approaches capable of improving patient stratification and guiding personalized management. In this context, liquid biopsy has emerged as a promising, minimally invasive approach able to capture tumor-derived molecular information through the analysis of circulating cell-free nucleic acids, including circulating cell-free DNA (cfDNA) and circulating cell-free RNA (cfRNA). Released into the bloodstream by tumor cells, these analytes offer a real-time and comprehensive snapshot of tumor biology, capturing genetic, epigenetic, and transcriptional alterations through a simple blood draw. Liquid biopsy-based analyses hold significant potential for early detection, prognostic assessment, therapeutic decision-making, monitoring of minimal residual disease, and identification of resistance mechanisms. This review discusses the current state of research on circulating cell-free nucleic acids in PC, highlighting their biological basis, methodological approaches, clinical potential, and the challenges limiting their widespread implementation. By underscoring their translational relevance, we aim to outline how integrated liquid biopsy strategies, alongside the need for standardization and cross-study harmonization, may contribute to a more precise and dynamic approach to PC management.

## 1. Introduction

### 1.1. Pancreatic Cancer

Pancreatic cancer (PC) is a highly lethal malignancy with a persistently poor prognosis. Adenocarcinoma arising from the ductal system of the pancreas (i.e., pancreatic ductal adenocarcinoma) accounts for over 90% of all PC and is widely recognized as the most aggressive type of primary pancreatic neoplasm.

Despite advances in medical research and therapeutic strategies, the worldwide burden of PC continues to increase. Over the past few decades its epidemiology has evolved significantly, and although incidence rates vary considerably across countries, global trends indicate a steady rise in diagnoses. PC is therefore projected to become the second leading cause of cancer-related death in Western countries [1].

Surgical resection remains the only potentially curative treatment. However, at the time of diagnosis, only approximately 15–20% of cases are resectable, while 30–35% present as locally advanced but unresectable due to invasion of adjacent major blood vessels, and about 50–55% are metastatic [2]. Additionally, PC is characterized by poor responsiveness to standard systemic therapies, including FOLFIRINOX, NALIRIFOX and nab-paclitaxel/gemcitabine in advanced disease, as well as FOLFIRINOX, gemcitabine/capecitabine and gemcitabine monotherapy in the adjuvant post-surgical setting [3,4]. Consequently, the overall 5-year survival rates remain approximately 11%.

The etiopathogenesis of PC is a multi-step process driven by several biological events: (i) somatic genetic alterations; (ii) chronic inflammatory conditions such as chronic pancreatitis, which promote a pro-tumor environment; (iii) interactions within the tumor microenvironment; (iv) aberrant activation of signaling pathways, along with epigenetic modifications [5]. In addition, several risk factors contribute to PC development, including modifiable factors (e.g., smoking, alcohol misuse, obesity, dietary habits, and type 2 diabetes, especially recent-onset diabetes) and non-modifiable factors (e.g., gender, age, ethnicity, family history of PC, and inherited germline mutations) [6].

Due to its nonspecific clinical presentation, characterized by symptoms such as weight loss, abdominal pain, jaundice, and fatigue, PC is often diagnosed at an advanced stage. Diagnosis relies on a combination of clinical evaluation, medical imaging procedures (i.e., US: ultrasonography; EUS: endoscopic ultrasound; ERCP: endoscopic retrograde cholangiopancreatography; CT: computed tomography; MR: magnetic resonance), serum tumor marker evaluation, and pathological confirmation. The latter is typically based on cytological or histological examination of tissue specimens obtained via fine-needle aspiration (FNA) or fine-needle biopsy (FNB) [7,8,9]. The most widely used serum biomarker is carbohydrate antigen 19-9 (CA 19-9), which is elevated in approximatively 80% of PC and is commonly used to assess disease burden, monitor treatment response, and detect recurrence [10]. Pre-operative serum CA 19-9 levels ≥ 500 IU/mL may suggest disseminated disease and are associated with poorer post-operative prognosis, thus limiting the appropriateness of major surgical intervention in such cases [11]. However, CA 19-9 lacks diagnostic specificity, as it can also be elevated in benign conditions like cholangitis or pancreatitis, and it is not expressed in Lewis antigen-negative individuals (5–10% of the population), thereby limiting its clinical utility. Carcinoembryonic antigen (CEA) is another nonspecific marker that may be elevated in PC, although it is generally less informative in clinical practice [12]. Pathological confirmation on tissue samples can be challenging, as pancreatic tumors are often difficult to access. Moreover, tissue sampling through US-guided biopsy or EUS-guided FNA/FNB frequently yields specimens with low-cellularity or insufficient material, resulting in nondiagnostic or inconclusive findings and limiting the feasibility of repeated sampling for disease monitoring [13].

In this “scenario”, relying solely on imaging and serum tumor marker evaluations may limit accurate prognostic stratification and effectiveness of treatment strategies.

### 1.2. Emerging Landscape of Liquid Biopsy Testing

Liquid biopsy is an innovative approach that examines tumor-released components found in bodily fluids, such as circulating tumor cells, circulating cell-free nucleic acids, and extracellular vesicles, and holds great potential for the identification of cancer-associated biomarkers [14]. Compared with conventional tissue biopsies and serum tumor marker assessments, liquid biopsy is less invasive and faster, enables the overcoming of tumor spatial heterogeneity, provides a dynamic representation of tumor evolution, and improves repeatability through longitudinal analysis of sequential samples collected at different time intervals during treatment and follow-up. The implementation of genetic information obtained from liquid biopsy into the clinical work-up of cancer patients offers several valuable applications, including early cancer detection, enhanced risk stratification of aggressive tumors with higher relapse potential, treatment selection based on molecular alterations, real-time monitoring of treatment effectiveness, detection of minimal residual disease, and detection of tumor evolution and resistance mechanisms [15].

In recent years, circulating cell-free nucleic acids, including circulating cell-free DNA (cfDNA) and circulating cell-free RNA (cfRNA), have gained considerable interest as biomarkers for improving early detection and personalized management of PC [16,17,18]. These nucleic acids are released into the bloodstream by tumor cells through processes such as apoptosis, necrosis, and active secretion, and are often transported via extracellular vesicles such as exosomes. Notably, cfDNA contains a tumor-derived fraction known as circulating tumor DNA (ctDNA), which can be comprehensively analyzed to characterize somatic mutations, epigenetic alterations, and structural changes, including copy number variations and genomic rearrangements [19]. Moreover, cfDNA concentration and fragmentomic features can be exploited for cancer detection and tissue-of-origin identification [20]. Meanwhile, cfRNA comprises both coding and non-coding RNA species. The non-coding RNAs include microRNAs (miRNAs), piwi-interacting RNAs (piRNAs), small nucleolar RNAs (snoRNAs), transfer RNAs (tRNAs), and long non-coding RNAs in both linear (lncRNAs) and circular forms (circRNAs). These molecules play key roles in gene regulation through post-transcriptional control, chromatin remodeling, and RNA processing. Their dysregulation is closely associated with cancer, as they can exert either tumor-promoting or tumor-suppressive functions by modulating complex regulatory networks [21,22,23,24,25,26,27].

Ongoing advances in sensitive detection technologies, including digital PCR, next-generation sequencing approaches, biosensors, and microfluidics/lab-on-a-chip, are rapidly advancing liquid biopsy research. These developments offer promising opportunities for providing non-invasive diagnostic and monitoring strategies in PC.

The aim of this review is to provide an updated overview of the advances in liquid biopsy applications in PC over the past decade, with a focus on circulating cell-free nucleic acids and their potential clinical utility in early detection, prognosis assessment, and prediction of therapeutic response.

## 2. Materials and Methods

### Search Strategy for Literature Review

This narrative review was conducted using the free PubMed database, covering the period from January 2014 to September 2025. Medical Subject Headings (MeSH) terms related to pancreatic cancer (“pancreatic cancer”, “pancreatic neoplasms”) and circulating nucleic acids (“circulating RNA”, “cell free RNA”, “circulating transcriptome”, “circulating cell free DNA”, “circulating tumor DNA”) were used. These terms were combined using the Boolean operators “OR” and “AND”.

**Figure 1:**
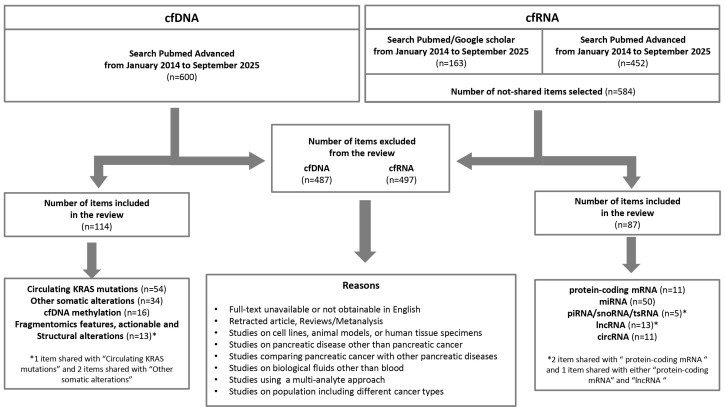
Flowchart illustrating the literature selection process for this review.

Inclusion criteria were predefined to ensure the selection of high-quality and relevant studies. Only original articles published in peer-reviewed journals, written in English, and available in full text within the specified timeframe were considered. Gray literature, including non-peer-reviewed or non-commercially published materials such as conference proceedings, theses, and institutional reports not indexed in major databases, was regarded as ineligible. Exclusion criteria included studies conducted on cell lines, animal models, or human tissue specimens; studies focused on pancreatic diseases other than PC; studies comparing PC with other pancreatic conditions. Additionally, to ensure methodological consistency and maintain a clear and coherent narrative while minimizing heterogeneity that could affect the interpretability and comparability of the findings, studies analyzing biological fluids other than blood, adopting a multi-analyte approach beyond circulating cell-free nucleic acids, or including populations with different cancer types were not considered. No restrictions were applied regarding the number of subjects or the methods employed for circulating cell-free nucleic acid quantification. For studies on miRNAs, only those reporting miRNAs identified in at least three independent studies were included. This threshold was adopted as a pragmatic criterion to prioritize biomarkers with a minimum level of reproducibility across independent investigations, thereby reducing the risk of overemphasizing findings from single reports (resulting in the exclusion of 22 articles) or from studies conducted by two independent groups with discordant results (resulting in the exclusion of 2 articles). The flowchart of the selection of the literature evaluated in this review is presented in Figure 1.

## 3. Results

### 3.1. Circulating Cell-Free DNA

#### 3.1.1. Circulating *KRAS* Mutations as Blood-Based Biomarkers

*KRAS*-mutated ctDNA is one of the most extensively investigated liquid biopsy approaches in PC. Its detection correlates with disease stage, tumor burden and prognostic stratification. Both baseline and on-treatment *KRAS* mutational burden may serve as dynamic biomarkers with prognostic and predictive value across all disease stages. This includes peri-operative and longitudinal monitoring of recurrence and survival in surgical candidates, potential support in selecting neoadjuvant therapy in borderline resectable disease (defined as PC with involvement of vascular structures, typically of the superior mesenteric vein/portal vein or arterial invasion), and assessment of treatment response in advanced and metastatic settings (Table 1). However, the strength of supporting evidence varies across studies and clinical contexts.

Quantitative metrics used to assess *KRAS*-mutated ctDNA, as well as *KRAS* mutation subtypes, may further refine prognostic stratification. Higher *KRAS* mutation concentrations correlate with both overall and progression-free survival, with defined cut-off values associated with worse clinical outcomes. For instance, a maximum variant allele frequency exceeding 10% is associated with decreased survival, whereas *KRAS* fractional abundance appears to correlate more closely with progression-free survival [28,29,30,31,32]. Additionally, increases in *KRAS* mutation concentration and fractional abundance levels at 6 months post-treatment are associated with decreased overall survival in patients with locally advanced and metastatic disease. The combination of *KRAS* mutation concentration and CA19-9 may further improve prognostic assessment [29]. Variant-specific effects are reported. Codon 12 mutations (G12V, G12C and G12D) are associated with poorer survival outcomes and increased risk of early recurrence in localized disease, particularly in minimal residual disease and post-resection settings [33,34,35,36,37]. Furthermore, cfDNA fragmentation patterns may provide additional refinement, as shorter cfDNA fragments harboring *KRAS* codon 12 hotspot mutations are detected in early-stage PC [38].

In resectable and borderline resectable PC, peri-operative assessment of *KRAS* ctDNA may provide prognostic and predictive information beyond CA 19-9. In patients undergoing pancreatic resection, Watanabe et al. identify a CA 19-9 threshold of 949.7 U/mL associated with the presence of *KRAS*-mutated ctDNA and poorer pre-operative prognosis [39]. Pre-operative detection of plasma *KRAS* may also predict post-operative recurrence and reduced overall and progression-free survival across several studies [28,29,30,31,40,41,42,43,44,45,46,47]. Post-operatively, the persistence or emergence of *KRAS*-mutated ctDNA is associated with early recurrence, often preceding radiologic evidence of progression, as well as an increased risk of liver metastases and worse clinical outcomes [28,31,36,45,46,48,49,50,51]. Longitudinal monitoring during adjuvant therapy suggests that rising *KRAS* ctDNA levels may identify patients at higher risk of early relapse and reduced overall survival [31,46,52,53]. Intra-operatively, detection of *KRAS* ctDNA after tumor resection may reflect surgery-related release of tumor DNA, while detection prior to tissue mobilization is associated with poorer overall and recurrence-free survival [50,54]. In borderline resectable and apparently localized disease, pre-operative ctDNA positivity is associated with increased risk of early recurrence and may help identify patients who could benefit from neoadjuvant therapy [55,56,57]. Following neoadjuvant chemotherapy, including modified FOLFIRINOX and gemcitabine plus nab-paclitaxel, post-operative *KRAS* ctDNA detection may predict poorer overall and progression-free survival, with potential additive prognostic value when combined with CA 19-9 [49,58,59].

In advanced PC, *KRAS* ctDNA detection is more frequently detected in metastatic compared with locally advanced disease and is often associated with liver or lung metastases, particularly in pre-chemotherapy settings [60,61,62]. The presence of *KRAS*-mutated ctDNA is associated with reduced overall survival across treatment modalities, whereas its absence or clearance during treatment is associated with improved response and disease control rates [28,30,60,63,64,65,66,67,68]. Baseline and on-treatment *KRAS* ctDNA levels may provide prognostic and predictive information, with early increases during chemotherapy associated with treatment resistance and disease progression [60,62,69,70,71,72]. Moreover, longitudinal monitoring may enable earlier detection of progression compared with CA 19-9 [28,61,64,65,66,73].

Multiple studies report higher levels of *KRAS*-mutated ctDNA in metastatic compared with localized disease, supporting its association with tumor burden [28,29,30,31,40,41,42,69]. *KRAS* ctDNA may also identify occult metastatic disease not evident on imaging, with improved performance when combined with CA 19-9 and CEA [55]. Clinical factors such as liver metastases and elevated CA 19-9 levels (≥2000 U/mL) are independently associated with a higher likelihood of detecting KRAS mutations [32]. In metastatic disease, *KRAS* ctDNA levels correlate with total tumor burden and liver metastatic volume, with higher mutation prevalence and allele frequency observed in patients with hepatic involvement [41,74]. Pre-treatment ctDNA positivity is associated with worse overall and disease-free survival, whereas early reductions after treatment initiation may indicate a favorable therapeutic response [41,75,76]. Prognostic stratification may be further enhanced by integrating *KRAS* mutation status with allele fraction, cfDNA fragmentation profiles, and CA 19-9 [76,77]. Variant-specific analyses suggest that detection of *KRAS* G12D during first-line chemotherapy may be associated with poorer outcomes, while its clearance may indicate treatment response [78,79]. Evidence from case reports further support the potential clinical utility of *KRAS* ctDNA monitoring, showing that ctDNA fluctuations may precede changes in CA 19-9 in identifying durable responses to gemcitabine combined with nab-paclitaxel in *ATM*-mutated metastatic PC [80].

**Table 1:** Summary of published studies on *KRAS*-mutant ctDNA in PC.

Year	PC Stage	Biological Source	Study Population(s)	Methodology	Clinical Significance	Ref.
2015	all stages	plasma	259 PC	ddPCR	prognosis	[42]
2015	all stages	serum	75 PC (discovery) 66 PC (validation) 20 HC	ddPCR	prognosis	[33]
2017	all stages	plasma	40 PC, 10 HC	ddPCR	prognosis	[34]
2018	all stages	plasma	77 PC	ddPCR	prognosis	[29]
2019	all stages	plasma	70 PC, 28 HC	SLHC-seq	early diagnosis prognosis	[38]
2019	all stages	plasma	78 PC	ddPCR	prognosis treatment response	[52]
2020	all stages	plasma	96 PC, 76 HC	ddPCR	prognosis	[40]
2020	all stages	plasma	135 PC	NGS	prognosis treatment response	[69]
2021	all stages	plasma	113 PC	ddPCR	prognosis	[39]
2021	all stages	plasma	72 PC	ddPCR	post-resection prognosis	[43]
2022	all stages	plasma	107 PC	ddPCR	prognosis	[41]
2023	all stages	plasma	108 PC	ddPCR	prognosis	[31]
2024	all stages	plasma	128 PC	ddPCR	prognosis	[35]
2025	all stages	plasma	106 PC	NGS	prognosis	[32]
2025	all stages	plasma	419 PC	ddPCR	prognosis	[28]
2025	all stages	plasma	200 PC	ddPCR	prognosis	[30]
2015	R	plasma	51 PC	NGS	prognosis	[48]
2016	R	plasma	105 PC, 20 HC	ddPCR	prognosis	[47]
2018	R	serum	45 PC	PNA clamp PCR	prognosis	[51]
2019	R	plasma	42 PC	PCR-based-SafeSeqS	prognosis	[45]
2019	R	plasma	59 PC	ddPCR	prognosis treatment response	[46]
2020	R	plasma	113 PC (discovery) 44 (validation)	NGS	prognosis	[37]
2021	R	plasma	105 PC	real-time PCR	prognosis	[44]
2021	R	plasma	25 PC	ddPCR	prognosis treatment response	[53]
2024	R	plasma	34 PC	ddPCR NGS	prognosis	[54]
2024	R	plasma	298 PC	mPCR-based NGS	prognosis	[36]
2021	R, LA	plasma	71 PC, 34 HC	ddPCR	prognosis	[50]
2021	R, BR, LA	plasma	165 PC	ddPCR	occult metastases prognosis	[55]
2023	R, BR, LA	plasma	66 PC	ddPCR	prognosis	[49]
2021	R, BR	plasma	97 PC	ddPCR	prognosis	[56]
2024	R, BR	plasma	46 PC	WGS	prognosis treatment response	[58]
2025	BR, LA	plasma	743 PC	ddPCR	prognosis	[57]
2022	BR	plasma	27 PC	ddPCR	prognosis treatment response	[59]
2018	LA	plasma	65 PC, 20 HC	ddPCR	prognosis	[63]
2015	LA + M	plasma	30 PC	ARMS PCR	prognosis treatment response	[67]
2016	LA + M	plasma	14 PC, 29 HC	PNA clamp PCR	prognosis treatment response	[66]
2017	LA + M	plasma	60 PC	BEAMing dPCR	treatment response	[68]
2017	LA + M	plasma	27 PC	ddPCR	treatment response	[72]
2018	LA + M	plasma	54 PC	BEAMing dPCR	prognosis treatment response	[64]
2020	LA + M	serum	45 PC	ddPCR/NGS	prognosis treatment response	[62]
2021	LA + M	plasma	29 PC	RT-PCR	prognosis treatment response	[65]
2023	LA + M	plasma	79 PC, 29 HC	PNA clamp PCR	prognosis treatment response	[60]
2023	LA + M	plasma	65 PC	ddPCR	prognosis treatment response	[70]
2023	LA + M	plasma	61 PC	ddPCR	treatment response	[73]
2024	LA + M	plasma	93 PC	PASEA	prognosis treatment response	[61]
2024	LA + M	plasma	18 PC	ddPCR	treatment response	[71]
2018	M	plasma	17 PC	NGS	prognosis treatment response	[76]
2020	M	plasma	61 PC	BEAMing dPCR ddPCR	prognosis	[77]
2020	M	plasma	1 PC	ddPCR	treatment response	[80]
2022	M	plasma	70 PC	ddPCR	prognosis treatment response	[75]
2023	M	plasma	512 PC	NGS	prognosis treatment response	[74]
2024	M	plasma	45 PC	PCR capillary gel electrophoresis	prognosis	[78]
2024	M	plasma	200 PC	ddPCR	prognosis treatment response	[79]

PC: pancreatic cancer; HC: healthy controls; R: resectable; BR: borderline resectable; LA: locally advanced; M: metastatic. ddPCR: droplet digital polymerase chain reaction; SLHC-seq: single-strand library preparation and hybrid-capture sequencing; NGS: next-generation sequencing; PNA clamp PCR: peptide nucleic acid clamp polymerase chain reaction; PCR-based-SafeSeqS: polymerase chain reaction based on safe-sequencing system; mPCR-based NGS: multiplex polymerase chain reaction-based next-generation sequencing; WGS: whole-genome sequencing; ARMS PCR: amplification-refractory mutation system polymerase chain reaction; BEAMing dPCR: beads, emulsion, amplification, magnetics, digital polymerase chain reaction; RT-PCR: real-time polymerase chain reaction; PASEA: programmable enzyme-assisted selective exponential amplification.

#### 3.1.2. Other Clinically Informative Somatic Alterations

Beyond *KRAS*, ctDNA analysis enables detection of additional potentially informative somatic alterations (Table 2). The most frequently detected mutations include *TP53*, *CDKN2A*, and *SMAD4*, which are associated with higher tumor stage and elevated serum markers, such as CA 19-9, CEA, and alkaline phosphatase [81].

In resected PC, pre-operative ctDNA analysis frequently identifies *KRAS* and *TP53* mutations, which are associated with increased tumor burden and worse clinical outcomes [82,83,84,85]. Detection rates and variant allele fractions rise with tumor size and stage, while post-operative declines may reflect changes in tumor burden better than CA 19-9 and CEA [86].

In resectable and borderline resectable PC, pre-operative detection of *KRAS* and *TP53* mutations in ctDNA, together with alterations in *EGFR*, *MET*, *SMAD4*, *BRAF*, *GNAS*, and *PIK3CA*, may predict occult metastatic disease and poorer post-operative prognosis [87]. In the neoadjuvant setting, *KRAS* and *TP53* are the dominant altered genes prior to treatment, along with mutations in *APC*, *FBXW7*, *FGFR2*, and *PIK3CA*. These alterations tend to persist during tumor progression, accompanied by mutations in *GNAS* and *FGFR3*, while increasing variant allele frequencies are associated with disease progression and adverse outcomes [88]. Co-occurring alterations in *KRAS*, *TP53*, and DNA damage repair genes are frequently observed in patients receiving neoadjuvant-modified FOLFIRINOX. In this context, DNA repair alterations may be associated with improved disease-free survival, whereas *KRAS* mutations remain linked to poorer outcomes [89].

Across borderline resectable or locally advanced PC, recurrent mutations in *KRAS*, *TP53*, *STK11*, and *FGFR2* identify patients with poor tumor differentiation, earlier progression during chemotherapy, and shorter disease-free survival, including after secondary resection [90]. In patients treated with PD-1 blockade plus chemoradiotherapy, an early reduction > 50% in ctDNA variant allele frequencies in *KRAS*, *TP53* and other genes is associated with improved survival, higher response rates, and more favorable post-operation pathological stage [91].

In advanced disease, ctDNA analysis reveals a higher prevalence of *KRAS* and *TP53* alterations compared with localized disease [85,92]. KRAS mutations show greater concordance with metastatic tissue, and are associated with poor differentiation and elevated CA 19-9, while both *KRAS* and *TP53* ctDNA levels correlate with higher tumor burden and shorter overall and disease-free survival [85,92,93,94,95]. Longitudinal ctDNA monitoring may enable earlier detection of disease progression compared with imaging or CA 19-9, while clearance of *KRAS* and *TP53* mutations during therapy is associated with improved disease control, even in second-line settings [93,94]. In patients receiving first-line FOLFIRINOX, ctDNA profiling captures alterations in *KRAS*, *TP53*, *CDKN2A*, and *SMAD4*, with baseline levels reflecting tumor and metastatic burden, and longitudinal changes tracking treatment response [96]. Broader ctDNA profiling expands the detectable mutational landscape. Variation in allele frequencies and clonal dynamics may track tumor burden and often precede changes in CA 19-9 during progression and treatment resistance [97]. In the first-line setting, specific alterations may further refine risk stratification, including *KRAS* enrichment in liver metastases and in non-responders, *CCND2* alterations associated with shorter progression-free survival, and clearance of *KRAS* and *TP53* mutations predicting improved clinical outcomes [98]. Detection of germline or somatic *BRCA1/2* mutations predicts sensitivity to platinum-based chemotherapy and PARP inhibitors, while reversion mutations may indicate acquired resistance [99,100]. In targeted treatment settings, *KRAS* mutations are associated with both primary and acquired resistance to anti-HER2 therapy [101].

Finally, ctDNA profiling provides valuable insight into treatment dynamics. During MEK-targeted therapies, baseline alterations in *KRAS*, *TP53*, *CDKN2A*, and *ATM* are associated with tumor burden, while on-treatment changes reflect clinical response [102]. Case-level evidence further suggests durable responses to MEK inhibitor monotherapy in locally advanced PC harboring multiple MEK pathway-related alterations [92]. Longitudinal monitoring also enables early detection of resistance mechanisms, such as acquisition of *MAP2K1* mutations during disease progression [103].

In metastatic disease, mutations in *KRAS* and *TP53* genes remain the most frequently detected somatic alterations prior to treatment, followed by *CDKN2A*, *SMAD4*, *BRAF*, *BRCA2*, *MTOR*, *EPHA7*, and *CDK12* [104,105,106]. These alterations show correlation with reduced overall survival. Additional mutations in *ARID1A*, *APC* and *FBXW7* genes may also contribute to adverse prognosis [107,108].

Similarly, ctDNA profiling shows potential clinical utility in predicting disease dynamics during treatment. Across first-line regimens (nab-paclitaxel plus S-1 or gemcitabine plus nab-paclitaxel plus immune checkpoint inhibitors durvalumab/tremelimumab), *KRAS* and *TP53* are associated with reduced survival, particularly in patients with liver metastases [107,109]. *TP53* alterations detected prior to FOLFIRINOX initiation, including somatic mutations and the homozygous germline TP53 Pro72Arg variant, are associated with early progression and adverse survival outcomes [110], while *ERBB2* exon 17 mutations may predict overall survival in patients treated with nab-paclitaxel plus gemcitabine [111]. Longitudinally, changes in *KRAS* and *TP53* allele frequencies may allow earlier detection of progression compared with imaging and tumor markers in patients across different treatment lines [104,105,112,113]. Moreover, combined analysis of *KRAS*, *TP53*, *CDKN2A*, *SMAD4*, and *ARID1A* may enable detection of disease progression approximately two months earlier than conventional imaging or serum biomarkers in patients receiving nab-paclitaxel plus S-1 [107]. Persistence of these ctDNA alterations after chemotherapy may identify patients with poor prognosis and inferior response to FOLFIRINOX or gemcitabine plus nab-paclitaxel chemotherapy, exceeding the predictive value of CA 19-9 [114].

Several studies report that ctDNA *KRAS* and *TP53* mutations, as well as alterations in *CDKN2A* and *SMAD4*, are associated with the presence of liver metastases, with concordance between ctDNA-based and tissue-based analyses [81,106,107]. Broader mutational signatures, including *SMAD4*, *BRAF*, *APC*, *FBXW7*, and less frequently altered genes, are associated with metastatic distribution (liver, lung, peritoneum), increased tumor burden, elevated CA 19-9 levels, and limited response to treatment [108]. Recent studies also suggest a role for *KRAS*, *LAMA1*, *FGFR1*, *IFFO1*, and adaptive immune-related genes in the development of liver metastases [105].

**Table 2:** Summary of published studies on clinically relevant somatic mutations identified in ctDNA in PC.

Year	PC Stage	Biological Source	Study Population	Methodology	Gene(s)	Clinical Significance	Ref.
2017	all stages	plasma	135 PC	NGS/ddPCR	*KRAS*, *TP53*	prognosis	[85]
2019	all stages	plasma	112 PC	NGS	*KRAS*, *TP53*	prognosis	[92]
2021	all stages	plasma	48 PC	NGS	*TP53*	prognosis	[110]
2025	all stages	plasma	414 PC	NGS	*KRAS*, *TP53*, *CDKN2A*, *SMAD4*	prognosis	[81]
2020	R	plasma	27 PC	NGS	*KRAS*, *TP53*	prognosis	[86]
2021	R	plasma	14 PC, 4 HC	NGS	*KRAS*, *TP53*, *SMAD4*, *ALK*	prognosis	[83]
2024	R	plasma	33 PC	NGS	*KRAS*, *TP53*	prognosis	[82]
2024	R	plasma	81 PC	NGS	*KRAS*, *TP53*	prognosis	[84]
2024	R, BR	plasma	30 PC	NGS	*KRAS*, *TP53*, *APC*, *FBXW7*, *FGFR2*, *PIK3CA*, *GNAS*, *FGFR3*	prognosis	[88]
2025	R, BR	plasma	135 PC	NGS	*KRAS*, *TP53*, *EGFR*, *MET*, *SMAD4*, *BRAF*, *GNAS*, *PIK3CA*	prognosis	[87]
2023	BR	plasma	28 PC	Guardant 360	*KRAS*, *TP53*, *ATM*, *BRCA1/2*, *MLH1*	prognosis	[89]
2022	BR, LA	plasma	69 PC	NGS	*KRAS*, *TP53*, *STK1*, *FGFR2*	prognosis	[90]
2023	BR, LA	plasma	29 PC	NGS	*KRAS*, *TP53*, *DNMT3A*, *ASXL1*, *CDKN2A*, *DNMT1*, *EPHA7*, *FGFR4*, *TET2*	prognosis treatment response	[91]
2019	LA	plasma	1 PC	NGS	*KRAS*, *GNAS*, *NF1*	treatment response	[92]
2015	LA + M	plasma	32 PC	Guardant 360	*KRAS*, *TP53*, *ATM*, *CDKN2A*	treatment response	[102]
2018	LA + M	plasma	19 PC	NGS	*BRCA1/2*	treatment response	[100]
2019	LA + M	plasma	38 PC, 13 HC	NGS	*KRAS*, *TP53*, *CDKN2A*, *SMAD4*	prognosis treatment response	[96]
2020	LA + M	plasma	141 PC	NGS	*KRAS*, *TP53*	prognosis longitudinal monitoring	[94]
2020	LA + M	plasma	223 PC	NGS	*KRAS*, *TP53*	prognosis	[95]
2022	LA + M	plasma	104 PC	NGS	*KRAS*, *TP53*, *CCND2*, *BRCA1/2*, *ATM*, *SMAD4*	prognosis	[98]
2022	LA + M	plasma	7 PC, 5 HC	NGS	*KRAS*, *TP53*, *SMAD4*, *DKN2A*, *NRAS*, *HRAS*, *MTOR*, *ERBB2*, *EGFR*, *PBRM1*	longitudinal monitoring	[97]
2022	LA + M	plasma	16 PC	NGS	*KRAS*, *MAP2K1*	treatment response	[103]
2023	LA + M	plasma	56 PC, 60 HC	HYTEC-seq ddPCR	*KRAS*, *TP53*	prognosis longitudinal monitoring	[93]
2024	LA + M	plasma	702 PC	Guardant 360	*BRCA1/2*, *ATM*	treatment selection	[99]
2024	LA + M	plasma	36 PC	Guardant 360	*KRAS*	treatment response	[101]
2017	M	plasma	188 PC	NGS ddPCR	*KRAS*, *ERBB2*	treatment response	[111]
2017	M	plasma	20 PC	NGS ddPCR	*KRAS*, *TP53*	treatment response	[112]
2020	M	plasma	58 PC	NGS	*KRAS*, *TP53*, *SMAD4*, *BRAF*	prognosis	[106]
2020	M	plasma	104 PC	NGS	*KRAS*, *TP53*, *APC*, *FBXW7*, *GNAS*, *ERBB2*, *CTNNB1*, *MAP2K1*, *EGFR*, *SMAD4*, *BRAF*, *NRAS*, *PIK3CA*	prognosis	[108]
2022	M	plasma	35 PC	NGS	*KRAS*, *TP53*, *CDKN2A*, *SMAD4*	prognosis longitudinal monitoring	[104]
2023	M	plasma	174 PC	NGS	*KRAS*, *TP53*, *CDKN2A*, *SMAD4*	prognosis treatment selection	[109]
2024	M	plasma	43 PC	NGS	*KRAS*, *TP53*, *CDKN2A*, *SMAD4*, *ARID1A*	prognosis longitudinal monitoring	[107]
2024	M	plasma	80 PC	WES ddPCR	*KRAS*, *TP53*, *BRCA2*, *MTOR*, *EPHA7*, *CDK12*, *LAMA1*, *FGFR1*, *IFFO1*, *HLA-H*, *HLA-DRB1*, *TRBV6-7*	prognosis liver metastasis longitudinal monitoring	[105]
2025	M	plasma	53 PC	NGS	*KRAS*, *TP53*, *CDKN2A*, *SMAD4*	treatment response	[114]
2025	M	plasma	12 PC	NGS	*KRAS*, *TP53*	treatment response	[113]

PC: pancreatic cancer; HC: healthy controls; R: resectable; BR: borderline resectable; LA: locally advanced; M: metastatic. NGS: next-generation sequencing; ddPCR: droplet digital polymerase chain reaction; HYTEC-seq: hybridization- and tag-based error-corrected sequencing; WES: whole-exome sequencing.

#### 3.1.3. cfDNA Methylation Analysis for Diagnosis and Prognostic Stratification

Analysis of cfDNA methylation represents a promising liquid biopsy strategy for improving the diagnosis and the prognostic stratification of PC (Table 3).

Multiple cfDNA methylation markers and models demonstrate high sensitivity and specificity for diagnosis of PC, in some cases exceeding the performance of CA 19-9. Shinjo et al. identify five methylated genes in serum cfDNA using methyl-CpG-binding protein-based digital PCR [115]. Their findings suggest that the combination of KRAS mutation detection with methylation positivity in at least one gene may improve detection of PC and is associated with larger tumor size and higher incidence of liver metastases. Whole-genome cfDNA methylation analyses further identify distinct hyper- and hypomethylated CpG sites in intergenic regions with high specificity for PC detection [116]. Henriksen et al. develop a plasma cfDNA methylation-specific PCR-based diagnostic model that outperforms CA 19-9 across different disease stages [117].

High-throughput sequencing models based on 5-methylcytosine (5mC) and 5-hydroxymethylcytosine (5hmC) markers also show the ability to discriminate between early and advanced disease [118]. In addition, genome-wide methylation profiling identifies eight methylated genes as candidate markers for early PC diagnosis [119]. Machine learning approaches integrating cfDNA 5hmC profiles demonstrate promising performance in high-risk populations, such as patients with type 2 diabetes [120]. Targeted ultra-deep sequencing approaches identify a methylation-based diagnostic model based on tissue–plasma concordant hypermethylated regions that outperform mutation-based models, with additional improvement observed when combined with CA 19-9 [121]. The methylation status of tumor suppressor genes may also be used to monitor surgical response, and its combination with CA 19-9 levels may enhance early PC detection [122]. Broader methylation classifiers, including a 37-gene hydroxy-methylation model [123], the PDACatch 56-marker classifier [124], and a 120-marker methylation panel [125], show higher diagnostic performance than CA 19-9, particularly in early-stage disease. Moreover, integration of cfDNA methylation with fragmentomic features (copy number alterations, fragment size, mutation signatures) may further improve early detection, especially in patients with negative CA 19-9, absence of jaundice, and small tumors [126].

Beyond diagnosis, cfDNA methylation markers are also related to clinical outcomes. *BRCA1/2* promoter methylation in plasma cfDNA is associated with reduced overall survival in resectable PC, with *BRCA1* methylation also showing correlation with poorer outcomes in metastatic disease [127]. *NPTX2* methylation may predict overall survival, and its longitudinal dynamics may anticipate disease progression compared with imaging and CA 19-9 [128]. Post hoc analyses of the PRODIGE 35 and 37 trials report that methylated *HOXD8* and *POU4F1* are independent prognostic factors in metastatic PC [129]. More recently, tumor-agnostic models integrating cfDNA methylation with other cfDNA features (fragment size and end motifs) have estimated ctDNA burden and predict clinical outcomes in advanced PC [130].

**Table 3:** Summary of published studies on ctDNA methylation markers in PC.

Year	PC Stage	Biological Source	Study Population(s)	Methodology	Methylated Target(s)	Clinical Significance	Ref.
2020	all stages	serum	47 PC, 14 HC	MBD–ddPCR	*ADAMTS2*, *HOXA1*, *PCDH10*, *SEMA5A*, *SPSB4*	diagnosis	[115]
2025	all stages	plasma	35 PC, 10 HC	WGS/NGS	CpG sites in intergenic regions	diagnosis	[116]
2016	all stages	plasma	95 PC	Methylation-specific PCR	*BMP3*, *RASSF1A*, *BNC1*, *MESTv2*, *TFPI2*, *APC*, *SFRP1*, *SFRP2*	diagnosis	[117]
2020	all stages	plasma	72 PC, 136 HC	High-throughput sequencing	5mC/5hmC signals	diagnosis	[118]
2023	all stages	plasma	132 PC, 528 HC (training) 102 PC, 2048 HC (validation)	NGS	5hmC signals	early diagnosis	[120]
2024	all stages	plasma	255 PC, 209 HC	Ultra-deep targeted NGS	*KCNA3*, *PRRX*, *CCNA1*, *TRIM58*, *NR2F1-AS1*	early diagnosis	[121]
2024	all stages	plasma	43 PC, 20 HC	NGS	*RASSF1A*, *EYA2*, *ppENK*, *p16*, *NPTX2*	early diagnosis	[122]
2020	all stages	plasma	64 PC, 243 HC	NGS	37-gene 5hmc model	early diagnosis	[123]
2022	all stages	plasma	198 PC, 323 HC	Targeted methylation sequencing	56-marker PDACatch classifier	early diagnosis	[124]
2025	all stages	plasma	50 PC, 52 HC	MCTA-Seq	120-marker methylation panel	early diagnosis	[125]
2025	all stages	plasma	166 PC, 167 HC (training) 112 PC, 111 HC (validation) 198 PC, 200 HC (validation)	NGS	Methylation patterns, fragment size, copy number variations and mutational signatures	early diagnosis	[126]
2020	R	plasma	4 PC, 2 HC (screening) 238 PC, 250 HC (training) 101 PC, 107 HC (validation)	High-throughput sequencing	*TRIM73*, *FAM150A*, *EPB41L3*, *SIX3*, *MIR663*, *MAPT*, *LOC100128977*, *LOC100130148*	early diagnosis	[119]
2024	R, M	plasma	105 PC, 40 HC	Methylation-specific real-time PCR	*BRCA1/2*	prognosis	[127]
2025	LA + M	plasma	33 PC, 19 HC	NGS	Methylation patterns and cfDNA features (fragment size and end motifs)	prognosis	[130]
2023	M	plasma	44 PC	ddPCR	*NPTX2*	prognosis	[128]
2022	M	plasma	372 PC, 12 HC	Met-ddPCR	*HOXD8*, *POU4F1*	prognosis	[129]

PC: pancreatic cancer; HC: healthy controls; R: resectable; LA: locally advanced; M: metastatic. MBD–ddPCR: methyl-CpG-binding protein–droplet digital polymerase chain reaction; WGS: whole-genome sequencing; NGS: next-generation sequencing; MCTA-Seq: methylated CpG tandem amplification and sequencing; ddPCR: droplet digital polymerase chain reaction; Met-ddPCR: methylation-specific droplet digital polymerase chain reaction.

#### 3.1.4. Fragmentomic Features, Actionable Mutations and Structural Alterations

Analysis of cfDNA through fragmentomic features and the identification of actionable and structural genomic alterations provides additional layers of information with the potential to refine prognostic assessment and guide therapeutic decision-making in PC (Table 4).

Fragmentomic analysis focuses on cfDNA quantitative and structural properties, including total concentration, fragment size distribution, and fragmentation patterns. Higher cfDNA levels and shorter fragment size are associated with reduced overall and progression-free survival [131,132]. In addition, an elevated neutrophil-to-lymphocyte ratio correlates with increased cfDNA levels and worse clinical outcomes [131].

Notably, cfDNA sequencing identifies potentially targetable somatic mutations in several genes in PC patients with *KRAS* minor allele frequency ≥ 1% and in refractory metastatic PDAC following pancreaticoduodenectomy, chemotherapy, and/or radiation therapy [42,133,134]. In advanced PC, therapeutically relevant alterations include *KRAS*, *PIK3CA*, *ATM*, *EGFR*, *MYC*, and *BRCA1/2*, alongside *KRAS*, *CCND2*, *SMAD4*, and *TP53* associated with metastatic disease; additional alterations emerging at disease progression involve *EGFR*, *PIK3CA*, *RET*, *MET*, *BRCA1*, *PDGFRA*, *ERBB2*, and *FGFR2* genes [135]. Moreover, ctDNA-guided treatment shows clinical benefit, with responses to pembrolizumab in *MLH1*-mutant cases and to olaparib in *BRCA1*-mutant tumors [95]. Further drug-targetable alterations in *PTEN*, *BRCA2*, and *TSC2* are reported in metastatic disease [105]. Importantly, ctDNA-based detection of microsatellite instability-high (MSI-H) status in plasma may predict responsiveness to immunotherapy, with objective response rates up to 77% [136,137].

Finally, genome-wide cfDNA analyses enable detection of structural and copy number alterations. High ctDNA instability scores and increased tumor fraction are associated with shorter survival and higher tumor burden across disease stages [138,139]. Copy number alterations in KRAS are more frequent in metastatic than in locally advanced disease and correlate with worse clinical outcomes [140]. In PC with liver metastases, *KRAS* G12D amplification is associated with aggressive disease behavior and resistance to chemotherapy [141]. Copy number changes, including gains in *KRAS* and *MYC* and deletions in *TGFBR2* and *PBRM1*, correlate with chemotherapy response, while amplifications of *CCND1* and *ERBB2* further expand the spectrum of potentially relevant ctDNA-detectable copy number alterations in PC [34,101,139].

**Table 4:** Summary of published studies on cfDNA features and ctDNA actionable/structural alterations in PC.

Year	PC Stage	Biological Source	Study Population	Methodology	Alteration(s)	Clinical Significance	Refs.
2015	all stages	plasma	48 PC	NGS	*ALK*, *ATM*, *DNMT3A*, *EGFR*, *KIT*, *MAP2K4*, *PIK3CA*; copy number alterations (*CCND1*, *ERBB2*)	targetable alterations	[42]
2016	all stages	plasma	259 PC	NGS ddPCR	*KRAS*, *ALK*, *ATM*, *DNMT3A*, *EGFR*, *KIT*, *MAP2K4*, *PIK3CA*	targetable mutations	[133]
2020	all stages	plasma	70 PC	WGS	Tumor fraction; copy number alterations (*KRAS*, *MYC*, *TGFBR2*, *PBRM1*)	prognosis treatment response	[139]
2021	all stages	plasma	315 PC, 38 HC	WGS	Genomic instability	prognosis	[138]
2024	all stages	plasma	82 PC	Fluorometric	cfDNA concentration, neutrophil-to-lymphocyte ratio	prognosis	[131]
2020	LA	plasma	1 PC	Guardant 360	Microsatellite instability	treatment response	[137]
2018	LA + M	plasma	61 PC, 21 HC	Automated electrophoresis	cfDNA concentration, fragment size	prognosis	[132]
2019	LA + M	plasma	55 PC, 16 HC	WGS ddPCR	Copy number alteration (*KRAS*)	prognosis	[140]
2021	M	plasma	1 PC	ddPCR NGS	Copy number alteration (*KRAS*)	treatment response	[141]
2020	LA + M	plasma	2 PC	NGS	*MLH1*, *BRCA1*	treatment response	[95]
2021	LA + M	plasma	282 PC	NGS	*KRAS*, *PIK3CA*, *ATM*, *EGFR*, *MYC*, *BRCA1/2*, *CCND2*, *SMAD4*, *TP53*, *RET*, *MET*, *PDGFRA*, *ERBB2*, *FGFR2*	targetable mutations	[135]
2022	LA + M	plasma	10 PC	Guardant 360	Microsatellite instability	treatment response	[136]
2024	LA + M	plasma	36 PC	Guardant 360	Copy number alteration (*ERBB2*)	treatment response	[101]
2021	M	plasma	77 PC	Guardant 360	*BRCA2*, *STK11*, *KRAS*, *PIK3CA*, *ATM*, *NF-1*, *EGFR*, *FGFR*	targetable mutations	[134]
2024	M	plasma	80 PC	WES ddPCR	*PTEN*, *BRCA2*, *TSC2*	targetable mutations	[105]

PC: pancreatic cancer; HC: healthy controls; LA: locally advanced; M: metastatic. NGS: next-generation sequencing; ddPCR: droplet digital PCR; WGS: whole-genome sequencing; WES: whole-exome sequencing.

### 3.2. Circulating Cell-Free RNA

#### 3.2.1. Circulating Protein-Coding RNA Candidate Biomarkers

Accumulating evidence supports circulating mRNAs, particularly those associated with extracellular vesicles and exosomes, as potential diagnostic and prognostic biomarkers for PC (Table 5).

An early study by Yang et al. reports that serum *COL6A3* mRNA is elevated in PC, distinguishing patients from healthy controls and correlating with perineural invasion and smoking habit [142]. Subsequent research has shifted toward exosomal mRNAs, which exhibit greater stability and improved diagnostic performance. Among these, *GPC1* mRNA emerges as a diagnostic candidate. Nanoparticle-based assays detect elevated levels of serum exosomal *GPC1* mRNA in early-stage PC, with further increases observed during disease progression [143]. These findings are supported by combined-biomarker approaches. A dual-marker strategy integrating exosomal *GPC1* mRNA with macrovesicle-associated GPC1 protein may improve diagnostic capability compared with CA 19-9 alone and shows prognostic relevance in advanced disease [144]. Similarly, *WASF2* and *ARF6* mRNAs are upregulated in serum exosomes from PC patients and outperform CA19-9 in discriminating PC from healthy individuals, including early-stage disease [145]. Their combination with CA 19-9 may further improve diagnostic performance. Broader profiling studies identify panels of deregulated serum exosomal mRNAs associated with early diagnosis and tumor subtype differentiation. These include transcripts such as *PDX1*, *DCN*, and *CTSL*, which show subtype-specific expression patterns [146]. In addition, an eight-long-RNA vesicle-derived signature may improve diagnostic accuracy compared with CA 19-9 [147].

Computational approaches may further enhance PC detection. Analysis of datasets derived from the Gene Expression Omnibus (GEO) identifies a predictive model based on four RNA pairs (eight extracellular vesicle-derived RNAs) [148]. Meta-analyses of public RNA-seq datasets detect discriminative exosomal markers, including *HIST2H2AA3*, which correlates with *KRAS* mutation status [149].

Circulating mRNAs also show potential prognostic potential. Reduced plasma *EVL* mRNA levels are associated with advanced stage and poorer overall survival [150]. Multi-mRNA signatures derived from extracellular vesicles are also reported, including a nine-long-RNA panel associated with immune tumor microenvironment features and survival stratification [151], and a three-mRNA model that may enhance prognostic stratification when combined with tumor stage [152]. More recently, cfRNA profiling has revealed subtype-specific transcripts enriched in basal-like PDAC, which are associated with poor clinical outcomes [153].

For many of the aforementioned protein-coding genes, biological functions in PC are summarized in Supplementary Table S1.

**Table 5:** Circulating protein-coding RNAs deregulated in PC.

Year	mRNA	Biological Source(s)	Study Population(s)	Methodology	Clinical Significance	Ref.
2014	*COL6A3*	serum	44 PC, 30 HC	qRT-PCR	diagnosis	[142]
2017	*GPC1*	serum exosome	118 PC, 60 HC (discovery) 48 PC, 15 HC (validation)	LPHN–CHDC	early diagnosis	[143]
2024	*GPC1*	serum exosome	91 PC (discovery) 138 PC (non-blinded validation) 55 PC (blinded validation) 30 HC	ILN biochip	diagnosis prognosis	[144]
2018	*WASF2*, *ARF6*	serum exosome	27 PC, 13 HC	qRT-PCR	diagnosis	[145]
2020	*MMP8*, *TBX3*, *PDX1*, *CTSL*, *SIGLEC15*, *IL32*, *SIGLEC114*, *DCN*, *HOXA5*, *KLRB1*	serum exosome	2 PC, 2 HC	qRT-PCR	diagnosis	[146]
2020	*CLDN1*, *FGA*, *HIST1H2BK*, *ITIH2*, *KRT19*, *MARCH2*, *MAL2*, *TIMP1*	plasma extracellular vesicles	284 PC, 117 HC	ExLR-seq	diagnosis	[147]
2021	*FBXO7*, *MORF4L1*, *DDX17*, *TALDO1*, *AHNAK*, *TUBA1B*, *CD44*, *SETD3*	serum exosome	284 PC, 117 HC (training and testing; GSE133684) 44 PC, 27 HC (validation)	NGS qRT-PCR	diagnosis	[148]
2021	*HIST2H2AA3*, *LUZP6*, *HLA-DRA*	plasma exosome	14 PC, 32 HC (discovery; GSE100232, GSE100206) 284 PC, 117 HC (validation; GSE133684)	RNA-Seq	diagnosis	[149]
2021	*EVL*	plasma	79 PC, 19 HC	qRT-PCR	prognosis	[150]
2021	*ACD*, *ADK*, *CANT1*, *HAVCR2*, *LGALS9*, *LYL1*, *PKIG*, *TBL3*, *TP53I11*	plasma exosome	345 PC, 81 HC	ExLR-seq	prognosis	[151]
2023	*PPP1R12A*, *SCN7A*, *SGCD*	plasma exosome	65 PC (discovery) 91 PC (training) 83 PC (validation)	ddPCR	prognosis	[152]
2024	*DEGS1*, *KDELC1*, *RPL23AP7*	plasma/serum	14 PC (COMPASS), 12 PC (UK-Essen), 24 HC; 20 PC (COMPASS), 122 (NEOLAP), 24 HC	NGS RT-ddPCR	prognosis	[153]

PC: pancreatic cancer; HC: healthy controls. qRT-PCR: quantitative real-time polymerase chain reaction; LPHN–CHDC: lipid–polymer hybrid nanoparticles containing catalyzed hairpin DNA circuit; ILN biochip: immune lipoplex nanoparticle biochip; ExLR-seq: extracellular vesicle long RNA sequencing; NGS: next-generation sequencing; RNA-Seq: RNA sequencing; ddPCR: droplet digital polymerase chain reaction; RT-ddPCR: real-time droplet digital polymerase chain reaction.

#### 3.2.2. Circulating miRNAs and Other Small Non-Coding RNA Biomarkers and Signatures

##### MiRNA

MiRNAs fine-tune post-transcriptional gene expression by acting either alone or in concert with other miRNAs, forming complex regulatory networks that enable precise control of gene expression.

Single miRNA candidates in PC

MiRNAs are promising circulating biomarkers for the diagnosis and prognosis of PC. Table 6 summarizes miRNAs reported in at least three independent studies, whereas Supplementary Table S2 lists 12 additional miRNAs reported in two studies, all showing concordant results.

Among the most extensively studied miRNAs, miR-10b and miR-221 show consistent dysregulation across multiple biofluids. MiR-10b is significantly upregulated in plasma and plasma-derived exosomes. Its levels are associated with disease stage, discriminate between early and advanced disease, and normalize after surgical resection [154,155,156,157]. MiR-221 shows a more complex expression pattern, with increased levels in serum and plasma but reduced levels in plasma-derived exosomes. High levels of miR-221 are associated with lymph node involvement, distant metastasis, and chemotherapy resistance. In some settings, it outperforms CA 19-9 in discriminating metastatic disease [158,159,160,161,162].

MiR-196a is elevated in plasma and correlates with lymph node and distant metastasis. Its diagnostic performance improves in localized disease when overexpressed in exosomes [163,164,165].

MiR-1246 is increased in serum and in both serum- and plasma-derived exosomes. It shows high sensitivity for early-stage and precursor lesions such as IPMN. Its combination with CA 19-9 and CEA may further improve diagnostic sensitivity [165,166,167].

MiR-22 is upregulated in the serum and plasma, with plasma levels increasing with tumor stage; however, reduced plasma miR-22-3p levels, detected up to five years before diagnosis, are associated with increased PC risk in individuals aged ≥65 years [168,169,170].

MiR-25 is consistently elevated in the serum and plasma and outperforms CA 19-9 and CEA in early-stage diagnosis. Its diagnostic sensitivity improves when combined with CA 19-9 compared with CA 19-9 alone or CA 19-9/CA 125 panels [163,171,172].

MiR-192-5p is elevated in serum, plasma, and exosomes. It shows moderate diagnostic power and higher expression in advanced-stage PC [161,173,174].

MiR-21 is one of the most extensively studied biomarkers. Elevated levels in serum and plasma distinguish PC patients from healthy controls and other gastrointestinal cancers. Increased miR-21 levels are also associated with reduced overall survival and recurrence-free survival [161,163,175,176,177]. Serum exosomal miR-21 shows high sensitivity for early-stage detection and outperforms CEA, although CA 19-9 remains superior in advanced disease. Elevated serum exosomal miR-21 levels are associated with reduced survival and disease progression during chemotherapy [178]. Diagnostic accuracy for early-stage PC may further improve when plasma exosomal miR-21 is combined with miR-10b [156]. Moreover, plasma exosomal miR-21 levels decrease following surgical resection and correlate with advanced tumor stage, lymph node/liver metastasis, and shorter survival. It shows better diagnostic performance than CEA but lower than CA 19-9 [157,179,180]. Additionally, portal vein-derived plasma exosomal miR-21 is associated with tumor burden, pathological staging, lymphatic invasion, recurrence after surgery, and both overall and disease-free survival [180].

MiR-155 is dysregulated in PC, with increased levels in plasma, particularly in patients with lymph node metastasis, and reduced levels in serum. Elevated plasma exosomal miR-155 is associated with shorter disease-free survival and resistance to gemcitabine [163,164,168,181].

MiR-122 is consistently elevated in plasma, serum, and exosomes. It distinguishes PC patients from controls, and higher plasma miR-122-5p levels correlate with metastasis, advanced stage, and poor prognosis [162,174,182,183].

MiR-451a is increased in plasma and serum-derived exosomes. It distinguishes PC patients from healthy individuals and differentiates early-stage from advanced disease. It is. associated with lymphatic invasion, advanced stage, reduced recurrence-free survival after surgery, and worse overall clinical outcomes, including when measured in portal vein-derived plasma exosomes [178,180,184]. Higher levels in serum exosomes are also linked to reduced disease control rate during chemotherapy [178].

MiR-205 is elevated in serum and plasma exosomes and shows diagnostic and prognostic potential. When combined with CA 19-9, serum miR-205 may improve PC detection compared with CA 19-9 alone. Machine learning-based analyses further support its potential role in early detection, peri-operative assessment of tumor resection, and prognosis, with higher levels associated with disease progression and poorer survival [183,185,186].

MiR-1469 is elevated in plasma and serum and distinguishes patients from healthy controls. It is associated with lymph nodes and distant metastasis and with overall survival [187,188,189].

MiR-99a shows a variable pattern. It is increased in serum and downregulated in plasma. It shows good diagnostic performance, correlates with advanced stage, may predict early recurrence after surgical resection, and identifies patients with shorter post-operative outcomes [177,190,191].

The target genes and signaling pathways regulated by the above miRNAs are presented in Supplementary Table S3.

MiRNA panel candidates in PC

Analyses of blood-based miRNA panels provide evidence for several miRNA signatures with potential clinical utility in PC (Table 7). Girolimetti et al. show that integrating circulating and vesicle-associated miRNAs enables discrimination between PC patients and healthy controls [159]. Several models demonstrate improved diagnostic performance compared with individual miRNAs [186,188,192,193]. Moreover, comparative studies show that combinations of miRNAs often outperform CA 19-9 alone [194,195]. Importantly, combining miRNA signatures with CA 19-9 may further enhance diagnostic accuracy. This is shown for panels including miR-33a-3p and miR-320a [161], as well as for more complex indices integrating CA 19-9 with multi-miRNA signatures [196,197]. Recent findings also report combined circulating and exosomal miRNA signatures that, together with CA 19-9, enable detection of PC even in early-stage disease and in patients with CA 19-9-negative tumors [198].

**Table 6:** MicroRNAs with clinical significance in PC.

Year	miRNA	Biological Source(s)	Study Population(s)	Methodology	Regulation	Clinical Significance	Ref.
2014	miR-10b	plasma	17 PC, 20 HC	qRT-PCR	up	diagnosis	[154]
2015		plasma exosome	3 PC, 3 HC	LSPR-based quantification	up	diagnosis	[155]
2017		plasma exosome	29 PC, 6 HC	qRT-PCR	up	diagnosis	[157]
2020		plasma exosome	36 PC, 65 HC	Tethered cationic lipoplex nanoparticle biochip	up	diagnosis	[156]
2016	mir-221	serum	17 PC	qRT-PCR	up	treatment response	[158]
2018		plasma	87 PC, 48 HC	qRT-PCR	up	metastasis detection	[160]
2019		plasma	94 PC, 51 HC	qRT-PCR	up	diagnosis	[161]
2024		plasma/serum	9 PC, 4 HC	qRT-PCR	up	diagnosis	[159]
2018		plasma plasma exosome	20 PC, 10 HC (screening) 40 PC, 40 HC (training) 112 PC, 116 HC (testing) 64 PC, 64 HC (validation) 31 PC, 37 HC	qRT-PCR	up down	diagnosis	[162]
2016	miR-196a	plasma	76 PC, 82 HC (training) 82 PC, 88 HC (blinded validation) 10 PC, 90 HC (double-blinded validation)	qRT-PCR	up	diagnosis prognosis	[163]
2017		plasma exosome	15 PC, 15 HC	qRT-PCR	up	early diagnosis	[165]
2019		plasma	20 PC, 10 HC	qRT-PCR	up	diagnosis lymph node involvement	[164]
2015	miR-1246	serum exosome	131 PC, 30 HC	Microarray qRT-PCR	up	diagnosis	[167]
2017		plasma exosome	15 PC, 15 HC	qRT-PCR	up	early diagnosis	[165]
2020		serum	41 PC, 30 HC	qRT-PCR	up	diagnosis	[166]
2017	miR-22-3p	plasma	35 PC, 15 HC	qRT-PCR	up	early diagnosis	[169]
2021		serum	63 PC, 29 non-cancer controls, 34 non-PC (training) 25 PC, 81 non-PC (validation) 17 PC, 16 non-cancer controls (validation)	Microarray qRT-PCR	up	diagnosis	[168]
2024		plasma	185 PC, 185 HC (discovery) 277 PC, 277 HC (replication)	NanoString nCounter Human v3 miRNA Expression Assay	up	risk	[170]
2016	miR-25	serum	303 PC, 600 HC	qRT-PCR	up	diagnosis	[171]
2020		serum	80 PC, 91 HC	qRT-PCR	up	early diagnosis	[172]
2016		plasma	76 PC, 82 HC (training) 82 PC, 88 HC (blinded validation) 10 PC, 90 HC (double-blinded validation)	qRT-PCR	up	diagnosis	[163]
2020	miR-192-5p	serum exosome	44 PC, 12 HC	qRT-PCR	up	diagnosis	[173]
2021		serum	50 PC, 25 HC	qRT-PCR	up	diagnosis	[174]
2019		plasma	94 PC, 51 HC	qRT-PCR	up	diagnosis	[161]
2015	miR-21	plasma	32 PC, 30 HC	qRT-PCR	up	diagnosis prognosis	[179]
2016		serum	24 PC, 10 HC	qRT-PCR	up	diagnosis	[175]
2017		plasma exosome	29 PC, 6 HC	qRT-PCR	up	diagnosis	[157]
2018		serum exosome	32 PC, 22 HC	NGS qRT-PCR	up	early diagnosis prognosis chemoresistance	[178]
2018		serum	181 PC, 40 HC	qRT-PCR	up	diagnosis prognosis	[177]
2019		plasma exosome (PB/PVB)	55 PC, 20 HC	Microarray qRT-PCR	up	diagnosis prognosis	[180]
2020		plasma exosome	36 PC, 65 HC	Tethered cationic lipoplex nanoparticle biochip	up	diagnosis early diagnosis	[156]
2017		serum	56 PC, 15 HC	qRT-PCR	up	diagnosis	[176]
2016		plasma	76 PC, 82 HC (training) 82 PC, 88 HC (blinded validation) 10 PC, 90 HC (double-blinded validation)	qRT-PCR	up	diagnosis	[163]
2019		plasma	94 PC, 51 HC	qRT-PCR	up	diagnosis	[161]
2017	miR-155	plasma exosome	23 PC	qRT-PCR	up	prognosis	[181]
2019		plasma	20 PC, 10 HC	qRT-PCR	up	diagnosis lymph node involvement	[164]
2021		serum	63 PC, 29 non-cancer controls, 34 non-PC (training) 25 PC, 81 non-PC (validation) 17 PC, 16 non-cancer controls (validation)	Microarray qRT-PCR	down	diagnosis	[168]
2016		plasma	76 PC, 82 HC (training) 82 PC, 88 HC (blinded validation) 10 PC, 90 HC (double-blinded validation)	qRT-PCR	up	diagnosis prognosis	[163]
2020	miR-122-5p	plasma	5 PC, 5 HS (discovery) 112 PC, 150 HS (validation)	GeneChip miRNA 4.0 Array ddPCR	up	prognosis	[182]
2021		serum	50 PC, 25 HC	qRT-PCR	up	diagnosis	[174]
2022		plasma exosome	65 PC, 78 HC	qRT-PCR	up	diagnosis	[183]
2018		plasma plasma exosome	20 PC, 10 HC (screening) 40 PC, 40 HC (training) 112 PC, 116 HC (testing) 64 PC, 64 HC (validation) 31 PC, 37 HC	qRT-PCR	up	diagnosis	[162]
2018	miR-451a	plasma exosome	6 PC, 3 HC (discovery) 50 PC, 20 HC (validation)	Microarray qRT-PCR	up	prognosis	[184]
2019		plasma exosome (PB/PVB)	55 PC, 20 HC	Microarray qRT-PCR	up	diagnosis prognosis	[180]
2018		serum exosome	32 PC, 22 HC	NGS qRT-PCR	up	early diagnosis chemoresistance	[178]
2018	miR-205	serum	65 PC, 34 HC	qRT-PCR	up	diagnosis	[185]
2022		plasma exosome	65 PC, 78 HC	qRT-PCR	up	diagnosis prognosis	[183]
2023		serum	26 PC	NGS	up	prognosis	[186]
2020	miR-1469	serum	342 PC, 329 HC (discovery; GSE106817, GSE113486, GSE59856, GSE85589) 81 PC, 70 HC (validation; GSE112264, GSE124158)	Profiling by array	up	diagnosis	[188]
2020		serum	100 PC, 150 HC (GSE59856)	Profiling by array	up	diagnosis prognosis	[189]
2020		plasma	49 PC, 29 HC	qRT-PCR	up	diagnosis prognosis	[187]
2018	miR-99a	serum	181 PC, 40 HC	qRT-PCR	up	diagnosis	[177]
2019		serum	2 PC (screening) 26 PC (validation)	qRT-PCR	up	prognosis	[190]
2020		plasma	48 PC (discovery) 64 PC (validation)	NGS qRT-PCR	down	prognosis	[191]

PC: pancreatic cancer; HC: healthy controls. PB: peripheral blood; PVB: portal vein blood. qRT-PCR: quantitative real-time polymerase chain reaction; LSPR-based quantification: localized surface plasmon resonance-based quantification; NGS: next-generation sequencing.

MiRNA panels may also support the assessment of surgical response. The combined expression of miR-10b and let-7a distinguishes early- from late-stage PC and shows significant changes following surgical resection [199]. Similarly, serum miR-1290 and miR-1246 correlate with tumor stage and size, enhance diagnostic accuracy when combined with CA 19-9, and decrease after tumor resection [200].

Beyond diagnosis, different studies describe prognostic miRNA panels. A plasma-based three-miRNA score identifies patients with reduced survival [191]. Kandimalla et al. report a nine-miRNA serum signature associated with aggressive molecular subtypes and adverse outcomes, with improved survival prediction when combined with clinicopathological factors, even in pre-operative samples [201]. Nishiwada et al. identify an exosomal six-miRNA panel that may predict PC recurrence, with higher accuracy when combined with CA 19-9 compared with standard clinicopathological factors; this model is validated both before and after neoadjuvant therapy [202].

Finally, combined diagnostic–prognostic miRNA signatures are reported. A six-miRNA plasma panel distinguishes early- from late-stage PC with high sensitivity and correlates with overall survival [162]. In addition, elevated plasma miR-222-3p and miR-221-3p levels are associated with disease stage and reduced survival [203].

##### Other Small Non-Coding RNAs

Underrepresented classes of non-coding RNAs, including piRNAs, snoRNAs, and tRNAs, are also emerging as potential diagnostic biomarkers for PC (Table 8).

Kumar et al. identify ten deregulated piRNAs in serum-derived exosomes from PC patients, suggesting a role in exosome-mediated intercellular communication [146]. Plasma piR-162725 improves the diagnostic performance of CA 19-9 [204]. Saha et al. report eleven circulating piRNAs altered in PC, including piR-23246, piR-32858, and piR-9137. The concordant expression of these piRNAs in both plasma and tumor tissues further supports their biomarker potential [205].

Among snoRNAs, serum exosomal SNORA74A and SNORA25 are elevated in PC and show higher diagnostic accuracy than CA 19-9, particularly in early-stage disease [145].

Finally, five tRNAs are dysregulated in serum exosomes from PC patients [146], and two stable circulating tRNA-derived small RNAs show increased expression levels in PC and outperform CA 19-9 and CEA for diagnosis [206].

The biological functions regulated by the small non-coding RNAs described above are presented in Supplementary Table S4.

**Table 7:** MicroRNA signatures with clinical significance in PC.

Year	miRNA Signature	Biological Source(s)	Study Population(s)	Methodology	Regulation	Clinical Significance	Ref.
2024	*sEV-miR-155*, sEV-miR-21, sEV-miR-27a, miR-221-3p, miR-let-7a-5p; 23b-3p, miR-34a-5p, miR-193a-3p	plasma/serum (vesicle-associated and circulating); plasma	11 PC, 8 HC; 4 PC 5 HC	qRT-PCR ddPCR	*down*; up	diagnosis	[159]
2020	*miR-125a-3p*, miR-642b-3p, miR-5100	serum	342 PC, 329 HC (discovery) (GSE106817, GSE113486, GSE59856, GSE85589) 81 PC, 70 HC (validation) (GSE112264, GSE124158)	Profiling by array	*down*; up	diagnosis	[188]
2022	miR-125a-3p, miR-4530, miR-92a-2-5p	plasma	77 PC, 65 HC	qRT-PCR	up	diagnosis	[192]
2019	let-7b-5p, miR-192-5p, miR-19a-3p, miR-19b-3p, miR-223-3p, and miR-25-3p	serum/ serum exosome	159 PC, 137 HC; 32 PC, 32 HC	qRT-PCR	up	diagnosis	[193]
2023	miR-1246, miR-205-5p, miR-191-5p	serum	26 PC	NGS	up	diagnosis	[186]
2020	miR-181b, miR-196a, miR-210	plasma	40 PC, 40 HC	qRT-PCR	up	diagnosis	[194]
2014	miR-642b, miR-885-5p, miR-22	plasma	8 PC, 11 HC	Microarray qRT-PCR	up	diagnosis	[195]
2019	miR-33a-3p + miR-320a + CA 19-9	plasma	94 PC, 51 HC	qRT-PCR	up	diagnosis	[161]
2016	*miR-16*, *miR-27a*, *miR-25*, *miR-29c*, miR-483-5p, CA 19-9; *miR-16*, *miR-24*, *miR-27a*, *miR-30-5p*, *miR-323-3p*, miR-20a, *miR-25*, *miR-29c*, miR-483-5p, CA 19-9	serum	417 PC, 248 HC	qRT-PCR	*down*; up	diagnosis early stage	[196]
2014	index I: miR-145, *miR-150*, miR-223, *miR-636***;** index II: miR-26b, miR-34a, miR-122, miR-126star, miR-145, *miR-150*, miR-223, miR-505, *miR-636*, miR-885-5p	whole blood	409 PC, 312 HC	qRT-PCR	*down*; up	diagnosis early stage	[197]
2022	cf-miRNAs (miR-30c-5p, miR-340-5p, miR-335-5p, miR-23b-3p, miR-142-3p) + exo-miRNA candidates (miR-145-5p, miR-200b-3p, miR-429, miR-1260b, miR145-3p, miR-216b-5p, miR-200a-3p, miR-217-5p)	plasma/plasma exosome	44 PC, 57 HC (discovery) 124 PC, 67 HC (training/validation)	NGS qRT-PCR	up	diagnosis early diagnosis	[198]
2023	miR-10b, *miR-let7a*	plasma	90 PC, 60 HC (training) 15 PC, 2 HS (validation)	High-throughput nanoplasmonic quantification qRT-PCR	*down*; up	diagnosis early stage surgical response	[199]
2020	miR-1290, miR-1246+ CA 19-9	serum	120 PC, 40 HC	qRT-PCR	up	diagnosis surgical response	[200]
2020	*miR-99a-5p*, *miR-365a-3p*, miR-200c-3p	plasma	48 PC (discovery) 64 PC (validation)	NGS qRT-PCR	*down*; up	prognosis	[191]
2022	miR-205–5p, miR-934, *miR-192–5p*, *miR-194–5p*, *miR-194–3p*, *miR-215–5p*, *miR-375–3p*, *miR-552–3p*, *miR-1251–5p*	serum	279 (discovery (ICGC, TCGA) 51 PC (validation)	Microarray qRT-PCR	*down*; up	prognosis	[201]
2022	miR-130b-5p, miR-133a-3p, miR-195-5p, miR-432-5p, miR-1229-3p, miR-1273f	plasma/serum exosome	25 PC (discovery) 139 PC (training, pre-NAT validation) 46 PC (post-NAT validation)	WGS qRT-PCR	up	prognosis response to NAT	[202]
2018	miR-122-5p and miR-193b-3p, miR-221-3p and miR-125b-5p, miR-192-5p, miR-27b-3p	plasma	20 PC, 10 HC (screening) 40 PC, 40 HC (training) 112 PC, 116 HC (testing) 64 PC, 64 HC (validation)	qRT-PCR	up	diagnosis prognosis	[162]
2023	miR-222-3p, miR-221-3p	plasma	46 PC, 20 HC (training) 115+50 PC, 2759+40 HC (validation) (GSE106817, GSE112264)	qRT-PCR	up	diagnosis prognosis	[203]

PC: pancreatic cancer; HC: healthy controls. NAT: neoadjuvant therapy. qRT-PCR: quantitative real-time polymerase chain reaction; ddPCR: droplet digital polymerase chain reaction; NGS: next-generation sequencing; WGS: whole-genome sequencing. Downregulated miRNAs are reported in italics.

**Table 8:** Other circulating small non-coding RNAs detected in PC.

Year	sncRNA	Biological Source	Study Population	Methodology	Clinical Significance	Ref.
2020	piR-52959, piR-53108, piR-30690, piR-54479, piR-56621, piR-54888, piR-42185, piR-46410, piR-58897, piR-43043	serum exosomes	2 PC, 2 HC	qRT-PCR	diagnosis	[146]
2022	piR-162725	plasma	45 PC, 27 HC	NGS	diagnosis	[204]
2024	piR-32871, piR-28104, piR-32981 piR-32977, piR-1961, piR-32895, piR-32978, piR-775, piR-25274, piR-12654, piR-3411	plasma	15 PC, 16 HC	NGS	diagnosis	[205]
2019	SNORA74A, SNORA25, SNORA22, SNORA14B, SNORD22	serum exosomes	27 PC, 13 HC	qRT-PCR	diagnosis	[145]
2020	tRNA125-Thr-CGT, tRNA21-Ser-TGA, tRNA15-Cys-GCA, tRNA55-Ile-TAT tRNA5-Ile-TAT	serum exosomes	2 PC, 2 HC	qRT-PCR	diagnosis	[146]
2021	tRF-Pro-AGG-004, tRF-Leu-CAG-002	serum	30 PC, 30 HC	qRT-PCR	diagnosis	[206]

PC: pancreatic cancer; HC: healthy controls. qRT-PCR: quantitative real-time polymerase chain reaction; NGS: next-generation sequencing.

#### 3.2.3. Linear and Circular Long Non-Coding RNAs as Circulating Biomarkers

##### Linear Long Non-Coding RNA

Emerging evidence supports the potential clinical relevance of circulating free and exosome-derived lncRNAs as non-invasive diagnostic and prognostic biomarkers in PC (Table 9).

Plasma levels of ABHD11-AS1 exhibit strong diagnostic performance, outperforming currently used serum biomarkers, and show prognostic relevance based on The Cancer Genome Atlas (TCGA) analyses [207]. CRNDE and MALAT-1 are upregulated in serum exosomes [146]. Elevated serum UFC1 and exosomal UCA1 levels are associated with advanced disease, metastasis, and poor survival [208,209]. Elevated plasma exosomal Sox2ot levels correlate with advanced TNM stage and reduced survival. Notably, its Sox2ot levels decrease after tumor resection, and are implicated in promotion of epithelial–mesenchymal transition via Sox2 regulation [210].

HULC expression is increased in both serum and exosomes [211,212]. While some studies report no significant association with clinicopathological factors [211], others show concordance between HULC serum and tissue levels, and correlations with tumor size, TNM stage, vascular invasion, and poorer overall survival [212]. Similarly, HOTTIP-005 and RP11-567G11.1 are overexpressed in PC tissues and correlate with aggressive tumor features and poor prognosis, while their corresponding RNA fragments in plasma, namely HDRF and RDRF, exhibit diagnostic value and decrease after surgical resection [213]. In addition, SNHG15, HOTAIR, C9orf139, and LINC01232 are upregulated in tissue and serum samples and are associated with advanced tumor stage, lymph node metastasis, poor differentiation, and reduced survival [214,215,216,217]. Mechanistically, HOTAIR promotes cancer metabolism through regulation of HK2 [215], while C9orf139 enhances tumor growth via the miR-663a/Sox12 signaling pathway [216].

For many of the aforementioned lncRNAs, their biological roles in PC are summarized in Supplementary Table S5.

**Table 9:** Long non-coding RNAs with clinical significance in PC.

Year	lncRNA	Biological Source	Study Population	Methodology	Clinical Significance	Ref.
2020	MALAT-1, CRNDE	serum exosome	2 PC, 2 HC	qRT-PCR	diagnosis	[146]
2018	Sox2ot	plasma	61 PC, 20 HC	Microarray	prognosis	[210]
2015	HOTTIP-005, RP11-567G11.1 (HDRF, RDRF)	plasma	127 PC, 122 HC	qRT-PCR	diagnosis/prognosis	[213]
2018	SNGH15	serum	171 PC, 59 HC	qRT-PCR	diagnosis/prognosis	[214]
2019	ABHD11-AS1	plasma	15 PC, 15 HC	qRT-PCR	diagnosis/prognosis	[207]
2019	UCF1	serum	40 PC, 40 HC	qRT-PCR	diagnosis/prognosis	[208]
2019	HULC	serum	60 PC, 60 HC	qRT-PCR	diagnosis/prognosis	[212]
2019	HOTAIR	serum	78 PC, 30 HC	qRT-PCR	diagnosis/prognosis	[215]
2020	UCA1	serum exosome	46 PC, 16 HC	qRT-PCR	diagnosis/prognosis	[209]
2020	HULC	serum extracellular vesicles	20 PC, 21 HC	dPCR	diagnosis/prognosis	[211]
2020	C9orf139	serum	54 PC, 30 HC	qRT-PCR	diagnosis/prognosis	[216]
2021	LINC01232	serum	108 PC, 60 HC	qRT-PCR	diagnosis/prognosis	[217]

PC: pancreatic cancer; HC: healthy controls. qRT-PCR: quantitative real-time polymerase chain reaction; dPCR: digital polymerase chain reaction.

##### Circular RNA

Accumulating evidence supports the role of circRNAs as potential biomarkers for PC detection and disease progression, with consistent upregulation observed in patient blood, matched tumor tissues, and cancer cell lines (Table 10).

Plasma or plasma-derived exosomal circ_0006220, circ_0001666, circLDLRAD3, and five-circRNA panel may enhance diagnostic performance when combined with CA 19-9. Moreover, circ_0006220, circ_0001666, and circLDLRAD3 together with circ_0013587 are associated with advanced disease and invasive tumor features (i.e., tumor size, lymph node metastasis, venous and lymphatic invasion) [218,219,220,221,222].

Elevated levels of circPDE8A, circIARS, circZNF91, and circRNA_000684 in plasma or exosomes are associated with tumor progression, development of metastasis, chemoresistance, and poor prognosis [223,224,225,226]. Functional studies suggest that these molecules may contribute to oncogenic signaling pathways and miRNA regulation. Additionally, circ_001569 and circPDK1 exhibit both diagnostic and prognostic value in PC [227,228]. The biological functions regulated by the circRNAs described below are presented in Supplementary Table S6.

**Table 10:** Circular RNAs with clinical significance in PC.

Year	circRNA	Biological Source	Study Population	Methodology	Clinical Significance	Ref.
2017	circLDLRAD3	plasma	31 PC, 31 HC	qRT-PCR	diagnosis	[219]
2021	circ_0013587	plasma	30 PC, 30 HC	qRT-PCR	diagnosis	[220]
2021	circPDAC	serum	20 PC, 20 HC	ddPCR	diagnosis	[221]
2022	circ_0006220, circ_0001666	plasma exosome	62 PC, 62 HC	qRT-PCR	diagnosis	[218]
2024	circ_0060733, circ_0006117 circ_0064288, circ_0007895 circ_0007367	plasma	158 PC, 81 HC	qRT-PCR	diagnosis	[222]
2018	circPDE8A	plasma exosome	93 PC	qRT-PCR	prognosis	[223]
2018	circIARS	plasma exosome	40 PC	qRT-PCR	prognosis	[224]
2021	circZNF91	plasma exosome	40 PC	qRT-PCR	prognosis	[225]
2021	circRNA_000684	plasma	38 PC, 38 HC	qRT-PCR	prognosis	[226]
2021	circ_001569	plasma	97 PC, 71 HC	qRT-PCR	diagnosis, prognosis	[227]
2022	circPDK1	serum exosome	20 PC, 10 HC	qRT-PCR	diagnosis, prognosis	[228]

PC: pancreatic cancer; HC: healthy controls. qRT-PCR: quantitative real-time polymerase chain reaction.

## 4. Discussion

The evidence reviewed herein supports the potential of circulating cell-free nucleic acids as minimally invasive tools for clinical management of PC. However, these biomarker classes can be stratified into distinct tiers according to their clinical maturity, reflecting differences in the strength of evidence, level of validation, and incremental value over established clinical standards.

### 4.1. Circulating KRAS and Other Somatic Alterations

**Figure 2:**
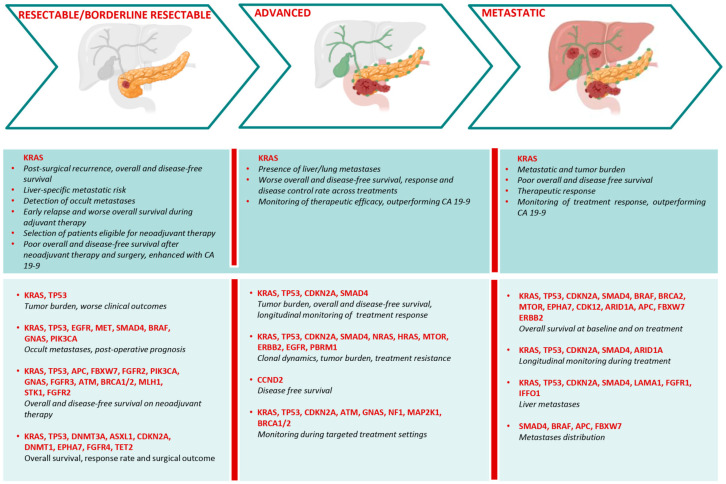
KRAS and other somatic mutations across pancreatic cancer stages: implications for clinical management.

The *KRAS* gene is a widely studied component of cfDNA, and its alterations are frequently accompanied by additional somatic alterations across different disease stages (Figure 2).

Multiple studies show associations between *KRAS*-mutated ctDNA and tumor burden, metastatic dissemination, molecular heterogeneity, and patient outcomes. Moreover, mutation subtypes may further refine prognostic stratification. Overall, *KRAS*-mutated ctDNA, both at baseline and during longitudinal monitoring, represents the most robust and clinically mature biomarker, supported by consistent evidence across disease stages and large patient cohorts. It provides reliable prognostic and predictive information, enables real-time monitoring of treatment response, and often detects disease progression earlier than conventional serum biomarkers such as CA 19-9 and standard imaging modalities. Despite these advantages, several limitations still restrict its routine clinical application. These include heterogeneity in assay methodologies, lack of standardized cut-offs used to define *KRAS* ctDNA positivity, absence of well-defined sampling intervals and intervention trials, variable concordance with tissue-based genotyping, limited comparative analyses between dynamics of *KRAS*-mutated ctDNA and conventional CA 19-9, and the need for large prospective validation studies.

Expanded ctDNA profiling reveals a complex mutational landscape that includes alterations in *TP53*, *CDKN2A*, and *SMAD4*, as well as less frequent alterations in DNA damage repair genes (*ATM*, *BRCA1*, *BRCA2*, *MLH1).* Across disease stages, higher ctDNA detection rates and variant allele frequencies are associated with larger tumor size, metastatic dissemination (particularly liver involvement), poorer differentiation, and worse survival outcomes. Longitudinal ctDNA profiling may outperform radiologic imaging and standard serum markers in detecting early disease progression, minimal residual disease, and acquired therapeutic resistance. Importantly, ctDNA-guided genomic profiling may also provide information on therapeutic sensitivity and resistance, including response to platinum-based chemotherapy, PARP inhibitors, MEK-targeted therapies, and anti-HER2 strategies in molecularly defined patient subsets. Despite these promising findings, the clinical applicability of these somatic alterations remains preliminary. Current findings are limited by small cohort sizes, heterogeneous study designs, and variability in analytical methods and platforms often based on non-standardized gene panels and cut-off values for ctDNA positivity. Furthermore, there is a lack of large prospective validation studies, and treatment selection data are derived from small cohorts or early-phase trials. As such, these findings should be considered hypothesis-generating rather than practice-changing and require confirmation in larger, well-defined studies with uniformly treated populations.

### 4.2. cfDNA Methylation, Fragmentomic Features, and Actionable/Structural Alterations

**Figure 3:**
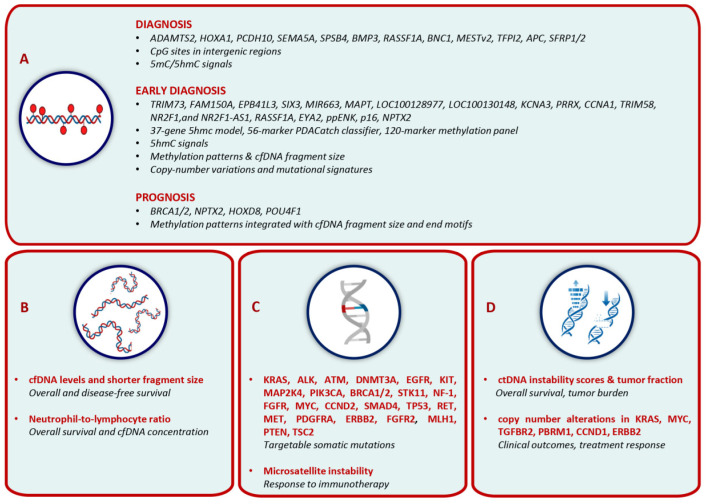
Liquid biopsy insights in pancreatic cancer: cfDNA methylation (**A**), fragmentomics/systemic inflammatory markers (**B**), actionable mutations (**C**), and structural alterations (**D**).

Additional evidence arises from the characterization of cfDNA methylation profiles and fragmentation patterns, which reflect the biological mechanisms underlying DNA release from tumors (including fragments size distribution, genomic positioning and end motifs), as well as from the identification of actionable mutations and structural genomic alterations in PC (Figure 3).

Methylation signatures in genes involved in tumor suppression, development, and differentiation show potential diagnostic value, with performance that may exceed CA 19-9, even in early-stage disease. Moreover, methylation of genes such as *BRCA1/2* and *NPTX2* may provide prognostic information and enable monitoring of disease dynamics, often preceding radiologic or serologic evidence of progression or treatment response. The identification of actionable alterations and acquired resistance mechanisms across disease stages may further inform the selection of targeted and immunotherapeutic strategies, including PARP inhibition and immune checkpoint blockade in molecularly defined subsets of patients. Additional cfDNA-based features, such as cfDNA concentration, shorter fragment size profiles, systemic inflammatory signatures, and copy number or structural alterations, may offer further prognostic and therapeutic insights. However, although cfDNA methylation classifiers and genomic instability metrics may enhance early detection, refine prognostic stratification and tumor burden assessment, and support therapeutic decision-making, their biological and clinical promise is still at a preliminary stage. In addition, their incremental value over established clinical tools is not yet clearly defined. Similarly, copy number alterations, emerging genomic variants, and single-gene methylation markers should still be considered exploratory, as current evidence is based on small, heterogeneous cohorts lacking robust validation. Finally, fragmentomics also remains of unclear clinical utility, limited by significant pre-analytical variability, lack of standardization, and unclear integration into current clinical practice. Likewise, systemic inflammatory markers such as the neutrophil-to-lymphocyte ratio have limited clinical applicability, as the available evidence is largely correlative rather than predictive.

### 4.3. CfRNA Alterations

**Figure 4:**
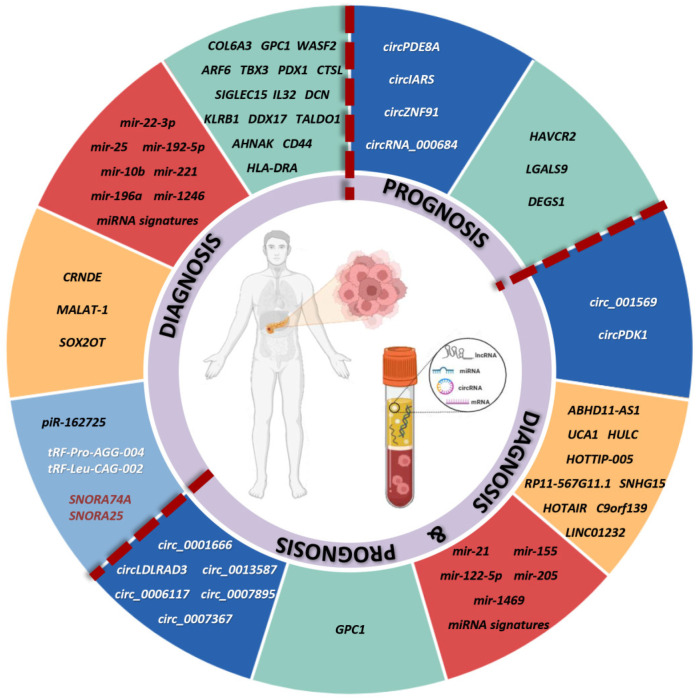
Circulating RNAs with clinical significance in pancreatic cancer. Different colors are used to represent the specific RNA classes: protein-coding RNAs (light green), microRNAs/microRNA signatures (red), long non-coding RNAs (orange), circular RNAs (blue), and other small non-coding RNAs (light blue).

CfRNA species may provide additional layers of biological information by capturing tumor biology, microenvironmental interactions, and transcriptional reprogramming (Figure 4).

cfRNAs encompass protein-coding mRNAs and multiple classes of non-coding RNAs (miRNAs, lncRNAs, circRNAs and other small RNAs), which differ substantially in their biological origin, stability, and potential clinical applications. A key distinction lies in their compartmentalization: while circulating mRNAs are characterized by a lower intrinsic stability, small non-coding RNAs, particularly miRNAs, exhibit high stability in circulation due to their association with protein complexes or vesicular encapsulation. These biological differences translate into distinct clinical niches.

#### 4.3.1. miRNAs

MiRNAs represent the most analytically robust class of cfRNAs, owing to their high stability in biofluids, resistance to RNase degradation, and reproducibility across different analytical platforms. Overall, miRNAs demonstrate broad clinical applicability in PC, ranging from early detection and disease staging to discrimination between localized and metastatic disease, often improving diagnostic accuracy when combined with CA 19-9. In addition, miRNAs provide prognostic and predictive information, enabling risk stratification, monitoring of treatment response and surgical outcomes, and identification of chemotherapy resistance and early disease recurrence. Importantly, miRNA panels integrate analytical robustness with improved diagnostic performance and reproducibility compared to a single miRNA, with enhanced diagnostic sensitivity in combination with established biomarkers. Nevertheless, despite their strong potential, variability in panel composition, normalization strategies, and assay platforms remains a major barrier to clinical standardization and widespread adoption.

#### 4.3.2. Protein-Coding RNA

On the other hand, mRNAs are considered promising components of multi-analyte signatures rather than standalone biomarkers for clinical use in PC. These molecules reflect active tumor transcriptional programs, may better capture tumor heterogeneity, and are increasingly explored for diagnostic and prognostic stratification and disease monitoring. However, their clinical implementation is limited by biological instability, greater susceptibility to pre-analytical variability, and the need for more complex isolation and sequencing workflows. In addition, current findings are often derived from relatively small or retrospective cohorts, and external validation remains limited.

#### 4.3.3. LncRNAs

LncRNAs also represent promising, albeit still preliminary, biomarkers. They reflect complex regulatory networks and may provide additional information on PC biology, supporting early diagnosis, prognostic stratification through associations with tumor stage, metastatic dissemination, and survival outcomes, as well as monitoring of surgical response. In addition, some lncRNAs may provide mechanistic insights into tumor progression and epithelial–mesenchymal transition. However, these molecules are currently better positioned as complementary biomarkers for prognosis and disease characterization rather than primary tools for early detection. Moreover, their clinical applicability remains limited by significant instability and the lack of standardized analytical methodologies.

#### 4.3.4. CircRNAs

CircRNAs are an emerging class of biomarkers whose unique covalently closed structure confers remarkable stability and resistance to degradation, thereby supporting their detection in circulation and extracellular vesicles. However, their clinical development remains at an early stage. In PC, circRNAs may have potential clinical applications ranging from early detection to prognostic stratification, particularly through associations with tumor invasiveness, metastatic dissemination, chemoresistance, and survival outcomes. Moreover, their integration with established biomarkers such as CA 19-9 may improve diagnosis and provide additional insights into oncogenic signaling pathways and disease progression.

CircRNAs act as regulatory molecules by sponging miRNAs, interacting with RNA-binding proteins, and modulating gene expression at multiple levels, thereby influencing tumor proliferation, invasion, metastasis, and treatment resistance. Increasingly, they are being recognized within the broader framework of gastrointestinal systems biology [229], where they participate in complex regulatory networks governing immune modulation, inflammatory signaling, metabolic reprogramming, and epithelial–mesenchymal transition. In addition, although not yet fully understood, potential interactions between circRNAs and the gut microbiome in shaping carcinogenesis and therapeutic response are emerging. This integrative role suggests that circRNAs may capture not only tumor-intrinsic alterations but also systemic and microenvironmental dynamics, thereby expanding their potential beyond conventional biomarkers.

However, despite their strong biological rationale, current evidence is largely derived from small, heterogeneous cohorts with limited validation and is further constrained by variability in detection methods and analytical pipelines. The complexity of circRNA regulatory interactions also poses challenges for standardization and clinical interpretation. As such, circRNAs should currently be considered promising but still investigational biomarkers, with potential applications pending robust validation and integration into multi-analyte biomarker strategies.

#### 4.3.5. piRNAs, snoRNAs, and tRNAs

Finally, despite promising early findings, other small non-coding RNAs presented in this review, including piRNAs, snoRNAs, and tRNAs, remain largely exploratory in the context of PC. Although they may prove useful for diagnosis and early detection, and several studies have reported diagnostic performance comparable or even superior to CA 19-9, their potential role as complementary non-invasive biomarkers in PC management still requires further validation. Most available studies are limited by small sample sizes, lack of independent validation cohorts, and incomplete understanding of the biological functions and dynamics of these molecules in circulation. Consequently, their current relevance lies primarily in hypothesis generation and mechanistic investigation rather than immediate clinical application.

## 5. Conclusive Remarks and Future Perspectives

Collectively, this evolving body of evidence underscores that, despite substantial advances, significant challenges continue to hinder the widespread clinical implementation of circulating cell-free nucleic acids in PC management. A central issue emerging from this review is the difficulty of cross-study interpretation, which represents a crucial bridge between laboratory discovery and clinical translation. Integrating data from independent studies offers the potential to enhance statistical power, reduce study-specific noise, and ultimately define robust, reproducible, and generalizable “consensus” molecular signatures across diverse patient populations and technological platforms. However, achieving such integration remains challenging. A major source of this complexity lies in the so-called batch effect, driven by heterogeneity in study design, including mixed disease stages, small cohorts, and underrepresentation of early-stage disease, as well as differences in biological matrices (plasma versus serum), pre-analytical handling, cfDNA and cfRNA isolation techniques, and exosome enrichment strategies. Additional variability arises from discrepancies in sequencing platforms, analytical thresholds, and bioinformatic pipelines. As a result, harmonizing these datasets requires sophisticated normalization strategies capable of minimizing technical variability while preserving true biological signals.

Another critical challenge is the lack of prospective head-to-head comparisons, a key step in translating biomarkers from discovery to clinical application. Such studies are essential to demonstrate the added value of novel biomarkers over established standards, including CA 19-9 and imaging modalities, yet remain largely absent.

Together, these hurdles constrain reproducibility and complicate the assessment of true clinical utility, meaning that many proposed biomarkers should still be regarded as exploratory despite encouraging early results. Moving forward, progress in the field will depend on the adoption of standardized workflows, the development of larger and stage-specific cohorts, and the execution of rigorously designed prospective studies. Particular emphasis should be placed on demonstrating clear incremental benefit over existing clinical tools and on validating integrative, multi-analyte approaches that combine genomic, epigenomic, and transcriptomic data. Concurrently, continued technological innovation is expected to accelerate progress and facilitate the translation of these approaches into clinical practice.

In conclusion, liquid biopsy strategies hold considerable promise for advancing PC management, but their successful integration into routine clinical practice will ultimately depend on overcoming these methodological and translational challenges.

## Data Availability

No new data were created or analyzed in this study.

## Supplementary Materials

The following supporting information can be downloaded at https://www.mdpi.com/article/10.3390/cells15100904/s1. Supplementary Tables. Table S1: Protein-coding RNAs with a biological role in pancreatic cancer. Table S2. MicroRNAs reported in two independent studies on pancreatic cancer with concordant findings. Table S3: MicroRNAs with a biological role in pancreatic cancer. Table S4: Other small non-coding RNAs with a biological role in pancreatic cancer. Table S5: Long non-coding RNAs with a biological role in pancreatic cancer. Table S6: Circular RNAs with a biological role in pancreatic cancer.

## Abbreviations

The following abbreviations are used in this manuscript:

ARMS PCR	Amplification-refractory mutation system polymerase chain reaction
BEAMing dPCR	Beads, emulsion, amplification, magnetics, digital polymerase chain reaction
BR	Borderline resectable
CA 19-9	Carbohydrate antigen 19-9
CEA	Carcinoembryonic antigen
cfDNA	Circulating cell-free DNA
cfRNA	Circulating cell-free RNA
circRNAs	Circular RNAs
CT	Computed tomography
ctDNA	Circulating tumor DNA
ddPCR	Droplet digital polymerase chain reaction
ERCP	Endoscopic retrograde cholangiopancreatography
EUS	Endoscopic ultrasound
FNA	Fine-needle aspiration
FNB	Fine-needle biopsy
HC	Healthy control
HYTEC-seq	Hybridization- and tag-based error-corrected sequencing
LA	Locally advanced
lncRNAs	Long non-coding RNAs
LSPR-based quantification	Localized surface plasmon resonance-based quantification
M	Metastatic
MBD–ddPCR	Methyl-CpG-binding protein–droplet digital polymerase chain reaction
MCTA-Seq	Methylated CpG tandem amplification and sequencing
Met-ddPCR	Methylation-specific droplet digital polymerase chain reaction
miRNAs	MicroRNAs
mPCR-based NGS	Multiplex polymerase chain reaction-based next-generation sequencing
MR	Magnetic resonance
NAT	Neoadjuvant therapy
NGS	Next-generation sequencing
PASEA	Programmable enzyme-assisted selective exponential amplification
PB	Peripheral blood
PC	Pancreatic cancer
PCR-based-SafeSeqS	Polymerase chain reaction based on safe-sequencing system
piRNAs	Piwi-interacting RNAs
PNA clamp PCR	Peptide nucleic acid clamp polymerase chain reaction
PVB	Portal vein blood
qRT-PCR	Quantitative real-time polymerase chain reaction
R	Resectable
RT-PCR	Real-time polymerase chain reaction
SLHC-seq	Single-strand library preparation and hybrid-capture sequencing
snoRNAs	Small nucleolar RNAs
tRNAs	Transfer RNAs
US	Ultrasonography
WES	Whole-exome sequencing
WGS	Whole-genome sequencing

## References

[1] Siegel R.L., Miller K.D., Wagle N.S., Jemal A. (2023). Cancer statistics, 2023. CA Cancer J. Clin..

[2] Park W., Chawla A., O’Reilly E.M. (2021). Pancreatic Cancer: A Review. JAMA.

[3] Conroy T., Pfeiffer P., Vilgrain V., Lamarca A., Seufferlein T., O’Reilly E.M., Hackert T., Golan T., Prager G., Haustermans K. (2023). Pancreatic cancer: ESMO Clinical Practice Guideline for diagnosis, treatment and follow-up. Ann. Oncol..

[4] Conroy T., Ducreux M., ESMO Guidelines Committee (2025). ESMO Clinical Practice Guideline Express Update on the management of metastatic pancreatic cancer. ESMO Open.

[5] Kleeff J., Korc M., Apte M., La Vecchia C., Johnson C.D., Biankin A.V., Neale R.E., Tempero M., Tuveson D.A., Hruban R.H. (2016). Pancreatic cancer. Nat. Rev. Dis. Primers.

[6] Olakowski M., Bułdak Ł. (2022). Modifiable and Non-Modifiable Risk Factors for the Development of Non-Hereditary Pancreatic Cancer. Medicina.

[7] Miura F., Takada T., Amano H., Yoshida M., Furui S., Takeshita K. (2006). Diagnosis of pancreatic cancer. HPB.

[8] Duffy M.J., Sturgeon C., Lamerz R., Haglund C., Holubec V.L., Klapdor R., Nicolini A., Topolcan O., Heinemann V. (2010). Tumor markers in pancreatic cancer: A European Group on Tumor Markers (EGTM) status report. Ann. Oncol..

[9] Zhao J., Wang J., Gu Y., Huang X., Wang L. (2025). Diagnostic methods for pancreatic cancer and their clinical applications (Review). Oncol. Lett..

[10] Luo G., Jin K., Deng S., Cheng H., Fan Z., Gong Y., Qian Y., Huang Q., Ni Q., Liu C. (2021). Roles of CA19-9 in pancreatic cancer: Biomarker, predictor and promoter. Biochim. Biophys. Acta Rev. Cancer.

[11] Isaji S., Mizuno S., Windsor J.A., Bassi C., Fernández-Del Castillo C., Hackert T., Hayasaki A., Katz M.H.G., Kim S.W., Kishiwada M. (2018). International consensus on definition and criteria of borderline resectable pancreatic ductal adenocarcinoma 2017. Pancreatology.

[12] Meng Q., Shi S., Liang C., Liang D., Xu W., Ji S., Zhang B., Ni Q., Xu J., Yu X. (2017). Diagnostic and prognostic value of carcinoembryonic antigen in pancreatic cancer: A systematic review and meta-analysis. OncoTargets Ther..

[13] Amaral M.J., Oliveira R.C., Donato P., Tralhão J.G. (2023). Pancreatic Cancer Biomarkers: Oncogenic Mutations, Tissue and Liquid Biopsies, and Radiomics—A Review. Dig. Dis. Sci..

[14] Marrugo-Ramírez J., Mir M., Samitier J. (2018). Blood-Based Cancer Biomarkers in Liquid Biopsy: A Promising Non-Invasive Alternative to Tissue Biopsy. Int. J. Mol. Sci..

[15] Pandey S., Yadav P. (2024). Liquid biopsy in cancer management: Integrating diagnostics and clinical applications. Pract. Lab. Med..

[16] Imamura T., Komatsu S., Ichikawa D., Kawaguchi T., Miyamae M., Okajima W., Ohashi T., Arita T., Konishi H., Shiozaki A. (2016). Liquid biopsy in patients with pancreatic cancer: Circulating tumor cells and cell-free nucleic acids. World J. Gastroenterol..

[17] McGowan R., Sally Á., McCabe A., Moran B.M., Finn K. (2022). Circulating Nucleic Acids as Novel Biomarkers for Pancreatic Ductal Adenocarcinoma. Cancers.

[18] Marin A.M., Sanchuki H.B.S., Namur G.N., Uno M., Zanette D.L., Aoki M.N. (2023). Circulating Cell-Free Nucleic Acids as Biomarkers for Diagnosis and Prognosis of Pancreatic Cancer. Biomedicines.

[19] Keller L., Belloum Y., Wikman H., Pantel K. (2021). Clinical relevance of blood-based ctDNA analysis: Mutation detection and beyond. Br. J. Cancer.

[20] Cristiano S., Leal A., Phallen J., Fiksel J., Adleff V., Bruhm D.C., Jensen S.Ø., Medina J.E., Hruban C., White J.R. (2019). Genome-wide cell-free DNA fragmentation in patients with cancer. Nature.

[21] O’Brien J., Hayder H., Zayed Y., Peng C. (2018). Overview of MicroRNA Biogenesis, Mechanisms of Actions, and Circulation. Front. Endocrinol..

[22] Svoronos A.A., Engelman D.M., Slack F.J. (2016). OncomiR or Tumor Suppressor? The Duplicity of MicroRNAs in Cancer. Cancer Res..

[23] Weng W., Li H., Goel A. (2019). Piwi-interacting RNAs (piRNAs) and cancer: Emerging biological concepts and potential clinical implications. Biochim. Biophys. Acta Rev. Cancer.

[24] Wang Y., Fu M., Zheng Z., Feng J., Zhang C. (2025). Small Nucleolar RNAs: Biological Functions and Diseases. MedComm.

[25] Berg M.D., Brandl C.J. (2021). Transfer RNAs: Diversity in form and function. RNA Biol..

[26] Chodurska B., Kunej T. (2025). Long non-coding RNAs in humans: Classification, genomic organization and function. Noncoding RNA Res..

[27] Conn V.M., Chinnaiyan A.M., Conn S.J. (2024). Circular RNA in cancer. Nat. Rev. Cancer.

[28] Chun J.W., Lee D.E., Kim M.K., Hwang J.H., Lee S.H., Jung M.K., Kim E.J., Ahn D.W., Kim Y.H., Han S.S. (2025). Optimal Value of Mutant KRAS Circulating Tumor DNA for Predicting Prognosis and Monitoring in Patients with Pancreatic Adenocarcinoma: A Prospective Multicenter Cohort Study. Clin. Chem..

[29] Kim M.K., Woo S.M., Park B., Yoon K.A., Kim Y.H., Joo J., Lee W.J., Han S.S., Park S.J., Kong S.Y. (2018). Prognostic Implications of Multiplex Detection of KRAS Mutations in Cell-Free DNA from Patients with Pancreatic Ductal Adenocarcinoma. Clin. Chem..

[30] Chun J.W., Lee D.E., Han N., Heo S., Kim H., Lee M.R., Park H.M., Han S.S., Park S.J., Kim T.H. (2025). Mutant KRAS and GATA6 Stratify Survival in Patients Treated with Chemotherapy for Pancreatic Adenocarcinoma: A Prospective Cohort Study. Cancers.

[31] Nitschke C., Markmann B., Walter P., Badbaran A., Tölle M., Kropidlowski J., Belloum Y., Goetz M.R., Bardenhagen J., Stern L. (2023). Peripheral and Portal Venous KRAS ctDNA Detection as Independent Prognostic Markers of Early Tumor Recurrence in Pancreatic Ductal Adenocarcinoma. Clin. Chem..

[32] Furukawa T., Fukada I., Hayashi N., Okamoto T., Sato Y., Maegawa Y., Hirai T., Mie T., Takeda T., Sasaki T. (2025). Clinical predictors of KRAS mutation detection in liquid biopsies for pancreatic ductal adenocarcinoma. Pancreatology.

[33] Kinugasa H., Nouso K., Miyahara K., Morimoto Y., Dohi C., Tsutsumi K., Kato H., Matsubara T., Okada H., Yamamoto K. (2015). Detection of K-ras gene mutation by liquid biopsy in patients with pancreatic cancer. Cancer.

[34] Ako S., Nouso K., Kinugasa H., Dohi C., Matushita H., Mizukawa S., Muro S., Akimoto Y., Uchida D., Tomoda T. (2017). Utility of serum DNA as a marker for KRAS mutations in pancreatic cancer tissue. Pancreatology.

[35] Lee M.R., Woo S.M., Kim M.K., Han S.S., Park S.J., Lee W.J., Lee D.E., Choi S.I., Choi W., Yoon K.A. (2024). Application of plasma circulating KRAS mutations as a predictive biomarker for targeted treatment of pancreatic cancer. Cancer Sci..

[36] Botta G.P., Abdelrahim M., Drengler R.L., Aushev V.N., Esmail A., Laliotis G., Brewer C.M., George G.V., Abbate S.M., Chandana S.R. (2024). Association of personalized and tumor-informed ctDNA with patient survival outcomes in pancreatic adenocarcinoma. Oncologist.

[37] Guo S., Shi X., Shen J., Gao S., Wang H., Shen S., Pan Y., Li B., Xu X., Shao Z. (2020). Preoperative detection of KRAS G12D mutation in ctDNA is a powerful predictor for early recurrence of resectable PDAC patients. Br. J. Cancer.

[38] Liu X., Liu L., Ji Y., Li C., Wei T., Yang X., Zhang Y., Cai X., Gao Y., Xu W. (2019). Enrichment of short mutant cell-free DNA fragments enhanced detection of pancreatic cancer. eBioMedicine.

[39] Watanabe F., Suzuki K., Tamaki S., Abe I., Endo Y., Takayama Y., Ishikawa H., Kakizawa N., Saito M., Futsuhara K. (2021). Optimal value of CA19-9 determined by KRAS-mutated circulating tumor DNA contributes to the prediction of prognosis in pancreatic cancer patients. Sci. Rep..

[40] Okada T., Mizukami Y., Ono Y., Sato H., Hayashi A., Kawabata H., Koizumi K., Masuda S., Teshima S., Takahashi K. (2020). Digital PCR-based plasma cell-free DNA mutation analysis for early-stage pancreatic tumor diagnosis and surveillance. J. Gastroenterol..

[41] Kirchweger P., Kupferthaler A., Burghofer J., Webersinke G., Jukic E., Schwendinger S., Weitzendorfer M., Petzer A., Függer R., Rumpold H. (2022). Circulating tumor DNA correlates with tumor burden and predicts outcome in pancreatic cancer irrespective of tumor stage. Eur. J. Surg. Oncol..

[42] Takai E., Totoki Y., Nakamura H., Morizane C., Nara S., Hama N., Suzuki M., Furukawa E., Kato M., Hayashi H. (2015). Clinical utility of circulating tumor DNA for molecular assessment in pancreatic cancer. Sci. Rep..

[43] Ako S., Kato H., Nouso K., Kinugasa H., Terasawa H., Matushita H., Takada S., Saragai Y., Mizukawa S., Muro S. (2021). Plasma KRAS mutations predict the early recurrence after surgical resection of pancreatic cancer. Cancer Biol. Ther..

[44] Li S., Zhang G., Li X., Li X., Chen X., Xu Y., Ren H. (2021). Role of the preoperative circulating tumor DNA KRAS mutation in patients with resectable pancreatic cancer. Pharmacogenomics.

[45] Lee B., Lipton L., Cohen J., Tie J., Javed A.A., Li L., Goldstein D., Burge M., Cooray P., Nagrial A. (2019). Circulating tumor DNA as a potential marker of adjuvant chemotherapy benefit following surgery for localized pancreatic cancer. Ann. Oncol..

[46] Groot V.P., Mosier S., Javed A.A., Teinor J.A., Gemenetzis G., Ding D., Haley L.M., Yu J., Burkhart R.A., Hasanain A. (2019). Circulating Tumor DNA as a Clinical Test in Resected Pancreatic Cancer. Clin. Cancer Res..

[47] Hadano N., Murakami Y., Uemura K., Hashimoto Y., Kondo N., Nakagawa N., Sueda T., Hiyama E. (2016). Prognostic value of circulating tumour DNA in patients undergoing curative resection for pancreatic cancer. Br. J. Cancer.

[48] Sausen M., Phallen J., Adleff V., Jones S., Leary R.J., Barrett M.T., Anagnostou V., Parpart-Li S., Murphy D., Kay Li Q. (2015). Clinical implications of genomic alterations in the tumour and circulation of pancreatic cancer patients. Nat. Commun..

[49] Hata T., Mizuma M., Motoi F., Ohtsuka H., Nakagawa K., Morikawa T., Unno M. (2023). Prognostic impact of postoperative circulating tumor DNA as a molecular minimal residual disease marker in patients with pancreatic cancer undergoing surgical resection. J. Hepato-Biliary-Pancreat. Sci..

[50] Hipp J., Hussung S., Timme-Bronsert S., Boerries M., Biesel E., Fichtner-Feigl S., Fritsch R., Wittel U.A. (2021). Perioperative cell-free mutant KRAS dynamics in patients with pancreatic cancer. Br. J. Surg..

[51] Nakano Y., Kitago M., Matsuda S., Nakamura Y., Fujita Y., Imai S., Shinoda M., Yagi H., Abe Y., Hibi T. (2018). KRAS mutations in cell-free DNA from preoperative and postoperative sera as a pancreatic cancer marker: A retrospective study. Br. J. Cancer.

[52] Watanabe F., Suzuki K., Tamaki S., Abe I., Endo Y., Takayama Y., Ishikawa H., Kakizawa N., Saito M., Futsuhara K. (2019). Longitudinal monitoring of KRAS-mutated circulating tumor DNA enables the prediction of prognosis and therapeutic responses in patients with pancreatic cancer. PLoS ONE.

[53] Hussung S., Akhoundova D., Hipp J., Follo M., Klar R.F.U., Philipp U., Scherer F., von Bubnoff N., Duyster J., Boerries M. (2021). Longitudinal analysis of cell-free mutated KRAS and CA 19-9 predicts survival following curative resection of pancreatic cancer. BMC Cancer.

[54] Maulat C., Canivet C., Cabarrou B., Pradines A., Selves J., Casanova A., Doussine A., Hanoun N., Cuellar E., Boulard P. (2024). Prognostic impact of circulating tumor DNA detection in portal and peripheral blood in resected pancreatic ductal adenocarcinoma patients. Sci. Rep..

[55] Hata T., Mizuma M., Iseki M., Takadate T., Ishida M., Nakagawa K., Hayashi H., Morikawa T., Motoi F., Unno M. (2021). Circulating tumor DNA as a predictive marker for occult metastases in pancreatic cancer patients with radiographically non-metastatic disease. J. Hepato-Biliary-Pancreat. Sci..

[56] Yamaguchi T., Uemura K., Murakami Y., Kondo N., Nakagawa N., Okada K., Seo S., Hiyama E., Takahashi S., Sueda T. (2021). Clinical Implications of Pre- and Postoperative Circulating Tumor DNA in Patients with Resected Pancreatic Ductal Adenocarcinoma. Ann. Surg. Oncol..

[57] Leiting J.L., Alva-Ruiz R., Yonkus J.A., Abdelrahman A.M., Lynch I.T., Carlson D.M., Carr R.M., Salomao D.R., McWilliams R.R., Starlinger P.P. (2025). Molecular KRAS ctDNA Predicts Metastases and Survival in Pancreatic Cancer: A Prospective Cohort Study. Ann. Surg. Oncol..

[58] Cecchini M., Salem R.R., Robert M., Czerniak S., Blaha O., Zelterman D., Rajaei M., Townsend J.P., Cai G., Chowdhury S. (2024). Perioperative Modified FOLFIRINOX for Resectable Pancreatic Cancer: A Nonrandomized Controlled Trial. JAMA Oncol..

[59] Kitahata Y., Kawai M., Hirono S., Okada K.I., Miyazawa M., Motobayashi H., Ueno M., Hayami S., Miyamoto A., Yamaue H. (2022). Circulating Tumor DNA as a Potential Prognostic Marker in Patients with Borderline-Resectable Pancreatic Cancer Undergoing Neoadjuvant Chemotherapy Followed by Pancreatectomy. Ann. Surg. Oncol..

[60] Edland K.H., Tjensvoll K., Oltedal S., Dalen I., Lapin M., Garresori H., Glenjen N., Gilje B., Nordgård O. (2023). Monitoring of circulating tumour DNA in advanced pancreatic ductal adenocarcinoma predicts clinical outcome and reveals disease progression earlier than radiological imaging. Mol. Oncol..

[61] Shen Y., Zhang X., Zhang L., Zhang Z., Lyu B., Lai Q., Li Q., Zhang Y., Ying J., Song J. (2024). Performance evaluation of a CRISPR Cas9-based selective exponential amplification assay for the detection of KRAS mutations in plasma of patients with advanced pancreatic cancer. J. Clin. Pathol..

[62] Sugimori M., Sugimori K., Tsuchiya H., Suzuki Y., Tsuyuki S., Kaneta Y., Hirotani A., Sanga K., Tozuka Y., Komiyama S. (2020). Quantitative monitoring of circulating tumor DNA in patients with advanced pancreatic cancer undergoing chemotherapy. Cancer Sci..

[63] Lin M., Alnaggar M., Liang S., Chen J., Xu K., Dong S., Du D., Niu L. (2018). Circulating Tumor DNA as a Sensitive Marker in Patients Undergoing Irreversible Electroporation for Pancreatic Cancer. Cell. Physiol. Biochem..

[64] Kruger S., Heinemann V., Ross C., Diehl F., Nagel D., Ormanns S., Liebmann S., Prinz-Bravin I., Westphalen C.B., Haas M. (2018). Repeated mutKRAS ctDNA measurements represent a novel and promising tool for early response prediction and therapy monitoring in advanced pancreatic cancer. Ann. Oncol..

[65] Schlick K., Markus S., Huemer F., Ratzinger L., Zaborsky N., Clemens H., Neureiter D., Neumayer B., Beate A.S., Florian S. (2021). Evaluation of circulating cell-free KRAS mutational status as a molecular monitoring tool in patients with pancreatic cancer. Pancreatology.

[66] Tjensvoll K., Lapin M., Buhl T., Oltedal S., Steen-Ottosen Berry K., Gilje B., Søreide J.A., Javle M., Nordgård O., Smaaland R. (2016). Clinical relevance of circulating KRAS mutated DNA in plasma from patients with advanced pancreatic cancer. Mol. Oncol..

[67] Semrad T., Barzi A., Lenz H.J., Hutchins I.M., Kim E.J., Gong I.Y., Tanaka M., Beckett L., Holland W., Burich R.A. (2015). Pharmacodynamic separation of gemcitabine and erlotinib in locally advanced or metastatic pancreatic cancer: Therapeutic and biomarker results. Int. J. Clin. Oncol..

[68] Van Laethem J.L., Riess H., Jassem J., Haas M., Martens U.M., Weekes C., Peeters M., Ross P., Bridgewater J., Melichar B. (2017). Phase I/II Study of Refametinib (BAY 86-9766) in Combination with Gemcitabine in Advanced Pancreatic cancer. Target. Oncol..

[69] Takada R., Ohkawa K., Kukita Y., Ikezawa K., Fukutake N., Abe Y., Imai T., Kiyota R., Nawa T., Yamai T. (2020). Clinical Utility of Pancreatic Cancer Circulating Tumor DNA in Predicting Disease Progression, Prognosis, and Response to Chemotherapy. Pancreas.

[70] Evrard C., Ingrand P., Rochelle T., Martel M., Tachon G., Flores N., Randrian V., Ferru A., Haineaux P.A., Goujon J.M. (2023). Circulating tumor DNA in unresectable pancreatic cancer is a strong predictor of first-line treatment efficacy: The KRASCIPANC prospective study. Dig. Liver Dis..

[71] Motobayashi H., Kitahata Y., Okada K.I., Miyazawa M., Ueno M., Hayami S., Miyamoto A., Shimizu A., Sato M., Yoshimura T. (2024). Short-term serial circulating tumor DNA assessment predicts therapeutic efficacy for patients with advanced pancreatic cancer. J. Cancer Res. Clin. Oncol..

[72] Del Re M., Vivaldi C., Rofi E., Vasile E., Miccoli M., Caparello C., d’Arienzo P.D., Fornaro L., Falcone A., Danesi R. (2017). Early changes in plasma DNA levels of mutant KRAS as a sensitive marker of response to chemotherapy in pancreatic cancer. Sci. Rep..

[73] Watanabe F., Suzuki K., Aizawa H., Endo Y., Takayama Y., Kakizawa N., Kato T., Noda H., Rikiyama T. (2023). Circulating tumor DNA in molecular assessment feasibly predicts early progression of pancreatic cancer that cannot be identified via initial imaging. Sci. Rep..

[74] Umemto K., Sunakawa Y., Ueno M., Furukawa M., Mizuno N., Sudo K., Kawamoto Y., Kajiwara T., Ohtsubo K., Okano N. (2023). Clinical significance of circulating-tumour DNA analysis by metastatic sites in pancreatic cancer. Br. J. Cancer.

[75] Kirchweger P., Kupferthaler A., Burghofer J., Webersinke G., Jukic E., Schwendinger S., Wundsam H., Biebl M., Petzer A., Rumpold H. (2022). Prediction of response to systemic treatment by kinetics of circulating tumor DNA in metastatic pancreatic cancer. Front. Oncol..

[76] Perets R., Greenberg O., Shentzer T., Semenisty V., Epelbaum R., Bick T., Sarji S., Ben-Izhak O., Sabo E., Hershkovitz D. (2018). Mutant KRAS Circulating Tumor DNA Is an Accurate Tool for Pancreatic Cancer Monitoring. Oncologist.

[77] Toledano-Fonseca M., Cano M.T., Inga E., Rodríguez-Alonso R., Gómez-España M.A., Guil-Luna S., Mena-Osuna R., de la Haba-Rodríguez J.R., Rodríguez-Ariza A., Aranda E. (2020). Circulating Cell-Free DNA-Based Liquid Biopsy Markers for the Non-Invasive Prognosis and Monitoring of Metastatic Pancreatic Cancer. Cancers.

[78] Hálková T., Bunganič B., Traboulsi E., Minárik M., Zavoral M., Benešová L. (2024). Prognostic Role of Specific KRAS Mutations Detected in Aspiration and Liquid Biopsies from Patients with Pancreatic Cancer. Genes.

[79] Till J.E., McDaniel L., Chang C., Long Q., Pfeiffer S.M., Lyman J.P., Padrón L.J., Maurer D.M., Yu J.X., Spencer C.N. (2024). Circulating KRAS G12D but not G12V is associated with survival in metastatic pancreatic ductal adenocarcinoma. Nat. Commun..

[80] Martino C., Pandya D., Lee R., Levy G., Lo T., Lobo S., Frank R.C. (2020). ATM-Mutated Pancreatic Cancer: Clinical and Molecular Response to Gemcitabine/Nab-Paclitaxel After Genome-Based Therapy Resistance. Pancreas.

[81] Keane F., Saadat L.V., O’Connor C.A., Chou J.F., Bowman A.S., Xu F., Crowley F., Debnath N., Schoenfeld J.D., Singhal A. (2025). Clinical utility and tissue concordance of circulating tumor DNA in pancreatic ductal adenocarcinoma. J. Natl. Cancer Inst..

[82] Imamura T., Ashida R., Urakami K., Ohshima K., Uesaka K., Sugiura T., Okamura Y., Ohgi K., Yamada M., Otsuka S. (2024). Comprehensive sequencing of circulating tumour DNA in resectable pancreatic cancer. Br. J. Surg..

[83] Affolter K.E., Hellwig S., Nix D.A., Bronner M.P., Thomas A., Fuertes C.L., Hamil C.L., Garrido-Laguna I., Scaife C.L., Mulvihill S.J. (2021). Detection of circulating tumor DNA without a tumor-informed search using next-generation sequencing is a prognostic biomarker in pancreatic ductal adenocarcinoma. Neoplasia.

[84] Theparee T., Akroush M., Sabatini L.M., Wang V., Mangold K.A., Joseph N., Stocker S.J., Freedman A., Helseth D.L., Talamonti M.S. (2024). Cell free DNA in patients with pancreatic adenocarcinoma: Clinicopathologic correlations. Sci. Rep..

[85] Pietrasz D., Pécuchet N., Garlan F., Didelot A., Dubreuil O., Doat S., Imbert-Bismut F., Karoui M., Vaillant J.C., Taly V. (2017). Plasma Circulating Tumor DNA in Pancreatic Cancer Patients Is a Prognostic Marker. Clin. Cancer Res..

[86] Jiang J., Ye S., Xu Y., Chang L., Hu X., Ru G., Guo Y., Yi X., Yang L., Huang D. (2020). Circulating Tumor DNA as a Potential Marker to Detect Minimal Residual Disease and Predict Recurrence in Pancreatic Cancer. Front. Oncol..

[87] Murakami T., Imamura M., Kimura Y., Watanabe K., Shinohara Y., Nakamura T., Low S.K., Motoya M., Kawakami Y., Masaki Y. (2025). Role of preoperative circulating tumor DNA in predicting occult metastases in resectable and borderline resectable pancreatic ductal adenocarcinoma. World J. Gastroenterol..

[88] Labiano I., Huerta A.E., Alsina M., Arasanz H., Castro N., Mendaza S., Lecumberri A., Gonzalez-Borja I., Guerrero-Setas D., Patiño-Garcia A. (2024). Building on the clinical applicability of ctDNA analysis in non-metastatic pancreatic ductal adenocarcinoma. Sci. Rep..

[89] Lim D.H., Yoon H., Kim K.P., Ryoo B.Y., Lee S.S., Park D.H., Song T.J., Hwang D.W., Lee J.H., Song K.B. (2023). Analysis of Plasma Circulating Tumor DNA in Borderline Resectable Pancreatic Cancer Treated with Neoadjuvant Modified FOLFIRINOX: Clinical Relevance of DNA Damage Repair Gene Alteration Detection. Cancer Res. Treat..

[90] Caliez O., Pietrasz D., Ksontini F., Doat S., Simon J.M., Vaillant J.C., Taly V., Laurent-Puig P., Bachet J.B. (2022). Circulating tumor DNA: A help to guide therapeutic strategy in patients with borderline and locally advanced pancreatic adenocarcinoma?. Dig. Liver Dis..

[91] Du J., Lu C., Mao L., Zhu Y., Kong W., Shen S., Tang M., Bao S., Cheng H., Li G. (2023). PD-1 blockade plus chemoradiotherapy as preoperative therapy for patients with BRPC/LAPC: A biomolecular exploratory, phase II trial. Cell Rep. Med..

[92] Patel H., Okamura R., Fanta P., Patel C., Lanman R.B., Raymond V.M., Kato S., Kurzrock R. (2019). Clinical correlates of blood-derived circulating tumor DNA in pancreatic cancer. J. Hematol. Oncol..

[93] Lapin M., Edland K.H., Tjensvoll K., Oltedal S., Austdal M., Garresori H., Rozenholc Y., Gilje B., Nordgård O. (2023). Comprehensive ctDNA Measurements Improve Prediction of Clinical Outcomes and Enable Dynamic Tracking of Disease Progression in Advanced Pancreatic Cancer. Clin. Cancer Res..

[94] Bachet J.B., Blons H., Hammel P., Hariry I.E., Portales F., Mineur L., Metges J.P., Mulot C., Bourreau C., Cain J. (2020). Circulating Tumor DNA is Prognostic and Potentially Predictive of Eryaspase Efficacy in Second-line in Patients with Advanced Pancreatic Adenocarcinoma. Clin. Cancer Res..

[95] Li H., Di Y., Li J., Jiang Y., He H., Yao L., Gu J., Lu J., Song J., Chen S. (2020). Blood-based Genomic Profiling of Circulating Tumor DNA from Patients with Advanced Pancreatic Cancer and its Value to Guide Clinical Treatment. J. Cancer.

[96] Wei T., Zhang Q., Li X., Su W., Li G., Ma T., Gao S., Lou J., Que R., Zheng L. (2019). Monitoring Tumor Burden in Response to FOLFIRINOX Chemotherapy Via Profiling Circulating Cell-Free DNA in Pancreatic Cancer. Mol. Cancer Ther..

[97] Sivapalan L., Thorn G.J., Gadaleta E., Kocher H.M., Ross-Adams H., Chelala C. (2022). Longitudinal profiling of circulating tumour DNA for tracking tumour dynamics in pancreatic cancer. BMC Cancer.

[98] Botrus G., Uson P.L.S., Raman P., Kaufman A.E., Kosiorek H., Yin J., Fu Y., Majeed U., Sonbol M.B., Ahn D.H. (2022). Circulating Cell-Free Tumor DNA in Advanced Pancreatic Adenocarcinoma Identifies Patients With Worse Overall Survival. Front. Oncol..

[99] Sudo K., Nakamura Y., Ueno M., Furukawa M., Mizuno N., Kawamoto Y., Okano N., Umemoto K., Asagi A., Ozaka M. (2024). Clinical utility of BRCA and ATM mutation status in circulating tumour DNA for treatment selection in advanced pancreatic cancer. Br. J. Cancer.

[100] Shroff R.T., Hendifar A., McWilliams R.R., Geva R., Epelbaum R., Rolfe L., Goble S., Lin K.K., Biankin A.V., Giordano H. (2018). Rucaparib Monotherapy in Patients With Pancreatic Cancer and a Known Deleterious BRCA Mutation. JCO Precis. Oncol..

[101] Barzi A., Weipert C.M., Espenschied C.R., Raymond V.M., Wang-Gillam A., Nezami M.A., Gordon E.J., Mahadevan D., Mody K. (2024). ERBB2 (HER2) amplifications and co-occurring KRAS alterations in the circulating cell-free DNA of pancreatic ductal adenocarcinoma patients and response to HER2 inhibition. Front. Oncol..

[102] Ko A.H., Bekaii-Saab T., Van Ziffle J., Mirzoeva O.M., Joseph N.M., Talasaz A., Kuhn P., Tempero M.A., Collisson E.A., Kelley R.K. (2016). Multicenter, Open-Label Phase II Clinical Trial of Combined MEK plus EGFR Inhibition for Chemotherapy-Refractory Advanced Pancreatic Adenocarcinoma. Clin. Cancer Res..

[103] Aung K.L., McWhirter E., Welch S., Wang L., Lovell S., Stayner L.A., Ali S., Malpage A., Makepeace B., Ramachandran M. (2022). A phase II trial of GSK2256098 and trametinib in patients with advanced pancreatic ductal adenocarcinoma. J. Gastrointest. Oncol..

[104] Guan S., Deng G., Sun J., Han Q., Lv Y., Xue T., Ding L., Yang T., Qian N., Dai G. (2022). Evaluation of circulating tumor DNA as a prognostic biomarker for metastatic pancreatic adenocarcinoma. Front. Oncol..

[105] Huerta M., Martín-Arana J., Gimeno-Valiente F., Carbonell-Asins J.A., García-Micó B., Martínez-Castedo B., Robledo-Yagüe F., Camblor D.G., Fleitas T., García Bartolomé M. (2024). ctDNA whole exome sequencing in pancreatic ductal adenocarcinoma unveils organ-dependent metastatic mechanisms and identifies actionable alterations in fast progressing patients. Transl. Res..

[106] Strijker M., Soer E.C., de Pastena M., Creemers A., Balduzzi A., Beagan J.J., Busch O.R., van Delden O.M., Halfwerk H., van Hooft J.E. (2020). Circulating tumor DNA quantity is related to tumor volume and both predict survival in metastatic pancreatic ductal adenocarcinoma. Int. J. Cancer.

[107] Huang L., Lv Y., Guan S., Yan H., Han L., Wang Z., Han Q., Dai G., Shi Y. (2024). High somatic mutations in circulating tumor DNA predict response of metastatic pancreatic ductal adenocarcinoma to first-line nab-paclitaxel plus S-1: Prospective study. J. Transl. Med..

[108] Uesato Y., Sasahira N., Ozaka M., Sasaki T., Takatsuki M., Zembutsu H. (2020). Evaluation of circulating tumor DNA as a biomarker in pancreatic cancer with liver metastasis. PLoS ONE.

[109] Renouf D.J., Loree J.M., Knox J.J., Topham J.T., Kavan P., Jonker D., Welch S., Couture F., Lemay F., Tehfe M. (2022). The CCTG PA.7 phase II trial of gemcitabine and nab-paclitaxel with or without durvalumab and tremelimumab as initial therapy in metastatic pancreatic ductal adenocarcinoma. Nat. Commun..

[110] van der Sijde F., Azmani Z., Besselink M.G., Bonsing B.A., de Groot J.W.B., Groot Koerkamp B., Haberkorn B.C.M., Homs M.Y.V., van IJcken W.F.J., Janssen Q.P. (2021). Circulating TP53 mutations are associated with early tumor progression and poor survival in pancreatic cancer patients treated with FOLFIRINOX. Ther. Adv. Med. Oncol..

[111] Cheng H., Liu C., Jiang J., Luo G., Lu Y., Jin K., Guo M., Zhang Z., Xu J., Liu L. (2017). Analysis of ctDNA to predict prognosis and monitor treatment responses in metastatic pancreatic cancer patients. Int. J. Cancer.

[112] Berger A.W., Schwerdel D., Ettrich T.J., Hann A., Schmidt S.A., Kleger A., Marienfeld R., Seufferlein T. (2017). Targeted deep sequencing of circulating tumor DNA in metastatic pancreatic cancer. Oncotarget.

[113] Christenson E.S., Lim S.J., Durham J., De Jesus-Acosta A., Bever K., Laheru D., Ryan A., Agarwal P., Scharpf R.B., Le D.T. (2022). Cell-free DNA Predicts Prolonged Response to Multi-agent Chemotherapy in Pancreatic Ductal Adenocarcinoma. Cancer Res. Commun..

[114] Kim H., Lee J., Park M.R., Choi Z., Han S.J., Kim D., Shin S., Lee S.T., Choi J.R., Park S.W. (2025). Prognostic Value of Residual Circulating Tumor DNA in Metastatic Pancreatic Ductal Adenocarcinoma. Ann. Lab. Med..

[115] Shinjo K., Hara K., Nagae G., Umeda T., Katsushima K., Suzuki M., Murofushi Y., Umezu Y., Takeuchi I., Takahashi S. (2020). A novel sensitive detection method for DNA methylation in circulating free DNA of pancreatic cancer. PLoS ONE.

[116] Ito T., Iwasawa T., Sakuraba S., Tanaka K. (2025). Plasma and Urine Circulating Tumor DNA Methylation Profiles for Non-Invasive Pancreatic Ductal Adenocarcinoma Detection: Significant Findings in Plasma Only. Int. J. Mol. Sci..

[117] Henriksen S.D., Madsen P.H., Larsen A.C., Johansen M.B., Drewes A.M., Pedersen I.S., Krarup H., Thorlacius-Ussing O. (2016). Cell-free DNA promoter hypermethylation in plasma as a diagnostic marker for pancreatic adenocarcinoma. Clin. Epigenetics.

[118] Cao F., Wei A., Hu X., He Y., Zhang J., Xia L., Tu K., Yuan J., Guo Z., Liu H. (2020). Integrated epigenetic biomarkers in circulating cell-free DNA as a robust classifier for pancreatic cancer. Clin. Epigenetics.

[119] Li S., Wang L., Zhao Q., Wang Z., Lu S., Kang Y., Jin G., Tian J. (2020). Genome-Wide Analysis of Cell-Free DNA Methylation Profiling for the Early Diagnosis of Pancreatic Cancer. Front. Genet..

[120] Haan D., Bergamaschi A., Friedl V., Guler G.D., Ning Y., Reggiardo R., Kesling M., Collins M., Gibb B., Hazen K. (2023). Epigenomic Blood-Based Early Detection of Pancreatic Cancer Employing Cell-Free DNA. Clin. Gastroenterol. Hepatol..

[121] Zhao G., Jiang R., Shi Y., Gao S., Wang D., Li Z., Zhou Y., Sun J., Wu W., Peng J. (2024). Circulating cell-free DNA methylation-based multi-omics analysis allows early diagnosis of pancreatic ductal adenocarcinoma. Mol. Oncol..

[122] Xin W., Tu S., Yi S., Xiong Y., Fang K., Sun G., Xiao W. (2024). Clinical significance of tumor suppressor genes methylation in circulating tumor DNA of patients with pancreatic cancer. Gene.

[123] Guler G.D., Ning Y., Ku C.J., Phillips T., McCarthy E., Ellison C.K., Bergamaschi A., Collin F., Lloyd P., Scott A. (2020). Detection of early stage pancreatic cancer using 5-hydroxymethylcytosine signatures in circulating cell free DNA. Nat. Commun..

[124] Wu H., Guo S., Liu X., Li Y., Su Z., He Q., Liu X., Zhang Z., Yu L., Shi X. (2022). Noninvasive detection of pancreatic ductal adenocarcinoma using the methylation signature of circulating tumour DNA. BMC Med..

[125] Hu W., Zhao X., Luo N., Xiao M., Feng F., An Y., Chen J., Rong L., Yang Y., Peng J. (2025). Circulating cell-free DNA methylation analysis of pancreatic cancer patients for early noninvasive diagnosis. Front. Oncol..

[126] Yin L., Cao C., Lin J., Wang Z., Peng Y., Zhang K., Xu C., Yang R., Zhu D., Wang F. (2025). Development and Validation of a Cell-Free DNA Fragmentomics-Based Model for Early Detection of Pancreatic Cancer. J. Clin. Oncol..

[127] Koukaki T., Balgkouranidou I., Biziota E., Karayiannakis A., Bolanaki H., Karamitrousis E., Zarogoulidis P., Deftereos S., Charalampidis C., Ioannidis A. (2024). Prognostic significance of BRCA1 and BRCA2 methylation status in circulating cell-free DNA of Pancreatic Cancer patients. J. Cancer.

[128] García-Ortiz M.V., Cano-Ramírez P., Toledano-Fonseca M., Cano M.T., Inga-Saavedra E., Rodríguez-Alonso R.M., Guil-Luna S., Gómez-España M.A., Rodríguez-Ariza A., Aranda E. (2023). Circulating NPTX2 methylation as a non-invasive biomarker for prognosis and monitoring of metastatic pancreatic cancer. Clin. Epigenetics.

[129] Pietrasz D., Wang-Renault S., Taieb J., Dahan L., Postel M., Durand-Labrunie J., Le Malicot K., Mulot C., Rinaldi Y., Phelip J.M. (2022). Prognostic value of circulating tumour DNA in metastatic pancreatic cancer patients: Post-hoc analyses of two clinical trials. Br. J. Cancer.

[130] Lapin M., Tjensvoll K., Edland K.H., Oltedal S., Garresori H., Gilje B., Ekedal S., Eftestøl T., Kvaløy J.T., Janku F. (2025). Tumor-agnostic detection of circulating tumor DNA in patients with advanced pancreatic cancer using targeted DNA methylation sequencing and cell-free DNA fragmentomics. Mol. Oncol..

[131] Varzaru B., Iacob R.A., Bunduc S., Manea I., Sorop A., Spiridon A., Chelaru R., Croitoru A., Topala M., Becheanu G. (2024). Prognostic Value of Circulating Cell-Free DNA Concentration and Neutrophil-to-Lymphocyte Ratio in Patients with Pancreatic Ductal Adenocarcinoma: A Prospective Cohort Study. Int. J. Mol. Sci..

[132] Lapin M., Oltedal S., Tjensvoll K., Buhl T., Smaaland R., Garresori H., Javle M., Glenjen N.I., Abelseth B.K., Gilje B. (2018). Fragment size and level of cell-free DNA provide prognostic information in patients with advanced pancreatic cancer. J. Transl. Med..

[133] Takai E., Totoki Y., Nakamura H., Kato M., Shibata T., Yachida S. (2016). Clinical Utility of Circulating Tumor DNA for Molecular Assessment and Precision Medicine in Pancreatic Cancer. Circulating Nucleic Acids in Serum and Plasma–CNAPS IX.

[134] Chung C., Galvin R., Achenbach E., Dziadkowiec O., Sen S. (2021). Characterization of Blood-Based Molecular Profiling in Pancreatic Adenocarcinoma. Oncology.

[135] Botrus G., Kosirorek H., Sonbol M.B., Kusne Y., Uson P.L.S., Borad M.J., Ahn D.H., Kasi P.M., Drusbosky L.M., Dada H. (2021). Circulating Tumor DNA-Based Testing and Actionable Findings in Patients with Advanced and Metastatic Pancreatic Adenocarcinoma. Oncologist.

[136] Chakrabarti S., Bucheit L., Starr J.S., Innis-Shelton R., Shergill A., Dada H., Resta R., Wagner S., Fei N., Kasi P.M. (2022). Detection of microsatellite instability-high (MSI-H) by liquid biopsy predicts robust and durable response to immunotherapy in patients with pancreatic cancer. J. Immunother. Cancer.

[137] Kamatham S., Shahjehan F., Kasi P.M. (2020). Circulating Tumor DNA-Based Detection of Microsatellite Instability and Response to Immunotherapy in Pancreatic Cancer. Front. Pharmacol..

[138] Woo S.M., Kim M.K., Park B., Cho E.H., Lee T.R., Ki C.S., Yoon K.A., Kim Y.H., Choi W., Kim D.Y. (2021). Genomic Instability of Circulating Tumor DNA as a Prognostic Marker for Pancreatic Cancer Survival: A Prospective Cohort Study. Cancers.

[139] Wei T., Zhang J., Li J., Chen Q., Zhi X., Tao W., Ma J., Yang J., Lou Y., Ma T. (2020). Genome-wide profiling of circulating tumor DNA depicts landscape of copy number alterations in pancreatic cancer with liver metastasis. Mol. Oncol..

[140] Mohan S., Ayub M., Rothwell D.G., Gulati S., Kilerci B., Hollebecque A., Sun Leong H., Smith N.K., Sahoo S., Descamps T. (2019). Analysis of circulating cell-free DNA identifies KRAS copy number gain and mutation as a novel prognostic marker in Pancreatic cancer. Sci. Rep..

[141] Pittella-Silva F., Kimura Y., Low S.K., Nakamura Y., Motoya M. (2021). Amplification of mutant KRASG12D in a patient with advanced metastatic pancreatic adenocarcinoma detected by liquid biopsy: A case report. Mol. Clin. Oncol..

[142] Kang C.Y., Wang J., Axell-House D., Soni P., Chu M.L., Chipitsyna G., Sarosiek K., Sendecki J., Hyslop T., Al-Zoubi M. (2014). Clinical significance of serum COL6A3 in pancreatic ductal adenocarcinoma. J. Gastrointest. Surg..

[143] Hu J., Sheng Y., Kwak K.J., Shi J., Yu B., Lee L.J. (2017). A signal-amplifiable biochip quantifies extracellular vesicle-associated RNAs for early cancer detection. Nat. Commun..

[144] Li H., Chiang C.L., Kwak K.J., Wang X., Doddi S., Ramanathan L.V., Cho S.M., Hou Y.C., Cheng T.S., Mo X. (2024). Extracellular Vesicular Analysis of Glypican 1 mRNA and Protein for Pancreatic Cancer Diagnosis and Prognosis. Adv. Sci..

[145] Kitagawa T., Taniuchi K., Tsuboi M., Sakaguchi M., Kohsaki T., Okabayashi T., Saibara T. (2019). Circulating pancreatic cancer exosomal RNAs for detection of pancreatic cancer. Mol. Oncol..

[146] Kumar S.R., Kimchi E.T., Manjunath Y., Gajagowni S., Stuckel A.J., Kaifi J.T. (2020). RNA cargos in extracellular vesicles derived from blood serum in pancreas associated conditions. Sci. Rep..

[147] Yu S., Li Y., Liao Z., Wang Z., Wang Z., Li Y., Qian L., Zhao J., Zong H., Kang B. (2020). Plasma extracellular vesicle long RNA profiling identifies a diagnostic signature for the detection of pancreatic ductal adenocarcinoma. Gut.

[148] Qin D., Zhao Y., Guo Q., Zhu S., Zhang S., Min L. (2021). Detection of Pancreatic Ductal Adenocarcinoma by A qPCR-based Normalizer-free Circulating Extracellular Vesicles RNA Signature. J. Cancer.

[149] Wu Y., Zeng H., Yu Q., Huang H., Fervers B., Chen Z.S., Lu L. (2021). A Circulating Exosome RNA Signature Is a Potential Diagnostic Marker for Pancreatic Cancer, a Systematic Study. Cancers.

[150] Du Y., Yao K., Feng Q., Mao F., Xin Z., Xu P., Yao J. (2021). Discovery and Validation of Circulating EVL mRNA as a Prognostic Biomarker in Pancreatic Cancer. J. Oncol..

[151] Li Y., Li Y., Yu S., Qian L., Chen K., Lai H., Zhang H., Li Y., Zhang Y., Gu S. (2021). Circulating EVs long RNA-based subtyping and deconvolution enable prediction of immunogenic signatures and clinical outcome for PDAC. Mol. Ther. Nucleic Acids.

[152] Han Y., Drobisch P., Krüger A., William D., Grützmann K., Böthig L., Polster H., Seifert L., Seifert A.M., Distler M. (2023). Plasma extracellular vesicle messenger RNA profiling identifies prognostic EV signature for non-invasive risk stratification for survival prediction of patients with pancreatic ductal adenocarcinoma. J. Hematol. Oncol..

[153] Metzenmacher M., Zaun G., Trajkovic-Arsic M., Cheung P., Reissig T.M., Schürmann H., von Neuhoff N., O’Kane G., Ramotar S., Dodd A. (2025). Minimally invasive determination of pancreatic ductal adenocarcinoma (PDAC) subtype by means of circulating cell-free RNA. Mol. Oncol..

[154] Ouyang H., Gore J., Deitz S., Korc M. (2014). microRNA-10b enhances pancreatic cancer cell invasion by suppressing TIP30 expression and promoting EGF and TGF-β actions. Oncogene.

[155] Joshi G.K., Deitz-McElyea S., Liyanage T., Lawrence K., Mali S., Sardar R., Korc M. (2015). Label-Free Nanoplasmonic-Based Short Noncoding RNA Sensing at Attomolar Concentrations Allows for Quantitative and Highly Specific Assay of MicroRNA-10b in Biological Fluids and Circulating Exosomes. ACS Nano.

[156] Pu X., Ding G., Wu M., Zhou S., Jia S., Cao L. (2020). Elevated expression of exosomal microRNA-21 as a potential biomarker for the early diagnosis of pancreatic cancer using a tethered cationic lipoplex nanoparticle biochip. Oncol. Lett..

[157] Lai X., Wang M., McElyea S.D., Sherman S., House M., Korc M. (2017). A microRNA signature in circulating exosomes is superior to exosomal glypican-1 levels for diagnosing pancreatic cancer. Cancer Lett..

[158] Tian X., Shivapurkar N., Wu Z., Hwang J.J., Pishvaian M.J., Weiner L.M., Ley L., Zhou D., Zhi X., Wellstein A. (2016). Circulating microRNA profile predicts disease progression in patients receiving second-line treatment of lapatinib and capecitabine for metastatic pancreatic cancer. Oncol. Lett..

[159] Girolimetti G., Pelisenco I.A., Eusebi L.H., Ricci C., Cavina B., Kurelac I., Verri T., Calcagnile M., Alifano P., Salvi A. (2024). Dysregulation of a Subset of Circulating and Vesicle-Associated miRNA in Pancreatic Cancer. Noncoding RNA.

[160] Li F., Xu J.W., Wang L., Liu H., Yan Y., Hu S.Y. (2018). MicroRNA-221-3p is up-regulated and serves as a potential biomarker in pancreatic cancer. Artif. Cells Nanomed. Biotechnol..

[161] Vila-Navarro E., Duran-Sanchon S., Vila-Casadesús M., Moreira L., Ginès À., Cuatrecasas M., Lozano J.J., Bujanda L., Castells A., Gironella M. (2019). Novel Circulating miRNA Signatures for Early Detection of Pancreatic Neoplasia. Clin. Transl. Gastroenterol..

[162] Zhou X., Lu Z., Wang T., Huang Z., Zhu W., Miao Y. (2018). Plasma miRNAs in diagnosis and prognosis of pancreatic cancer: A miRNA expression analysis. Gene.

[163] Yuan W., Tang W., Xie Y., Wang S., Chen Y., Qi J., Qiao Y., Ma J. (2016). New combined microRNA and protein plasmatic biomarker panel for pancreatic cancer. Oncotarget.

[164] Lemberger M., Loewenstein S., Lubezky N., Nizri E., Pasmanik-Chor M., Barazovsky E., Klausner J.M., Lahat G. (2019). MicroRNA profiling of pancreatic ductal adenocarcinoma (PDAC) reveals signature expression related to lymph node metastasis. Oncotarget.

[165] Xu Y.F., Hannafon B.N., Zhao Y.D., Postier R.G., Ding W.Q. (2017). Plasma exosome miR-196a and miR-1246 are potential indicators of localized pancreatic cancer. Oncotarget.

[166] Ishige F., Hoshino I., Iwatate Y., Chiba S., Arimitsu H., Yanagibashi H., Nagase H., Takayama W. (2020). MIR1246 in body fluids as a biomarker for pancreatic cancer. Sci. Rep..

[167] Madhavan B., Yue S., Galli U., Rana S., Gross W., Müller M., Giese N.A., Kalthoff H., Becker T., Büchler M.W. (2015). Combined evaluation of a panel of protein and miRNA serum-exosome biomarkers for pancreatic cancer diagnosis increases sensitivity and specificity. Int. J. Cancer.

[168] Lee J., Lee H.S., Park S.B., Kim C., Kim K., Jung D.E., Song S.Y. (2021). Identification of Circulating Serum miRNAs as Novel Biomarkers in Pancreatic Cancer Using a Penalized Algorithm. Int. J. Mol. Sci..

[169] Hussein N.A., Kholy Z.A., Anwar M.M., Ahmad M.A., Ahmad S.M. (2017). Plasma miR-22-3p, miR-642b-3p and miR-885-5p as diagnostic biomarkers for pancreatic cancer. J. Cancer Res. Clin. Oncol..

[170] Wang C., Cai H., Cai Q., Wu J., Stolzenberg-Solomon R., Guo X., Zhu C., Gao Y.T., Berlin J., Ye F. (2024). Circulating microRNAs in association with pancreatic cancer risk within 5  years. Int. J. Cancer.

[171] Deng T., Yuan Y., Zhang C., Zhang C., Yao W., Wang C., Liu R., Ba Y. (2016). Identification of Circulating MiR-25 as a Potential Biomarker for Pancreatic Cancer Diagnosis. Cell. Physiol. Biochem..

[172] Yu Y., Tong Y., Zhong A., Wang Y., Lu R., Guo L. (2020). Identification of Serum microRNA-25 as a novel biomarker for pancreatic cancer. Medicine.

[173] Flammang I., Reese M., Ströse A.J., Yang Z., Eble J.A., Dhayat S.A. (2020). Tumor-Suppressive miR-192-5p Has Prognostic Value in Pancreatic Ductal Adenocarcinoma. Cancers.

[174] Khan I.A., Rashid S., Singh N., Rashid S., Singh V., Gunjan D., Das P., Dash N.R., Pandey R.M., Chauhan S.S. (2021). Panel of serum miRNAs as potential non-invasive biomarkers for pancreatic ductal adenocarcinoma. Sci. Rep..

[175] Alemar B., Izetti P., Gregório C., Macedo G.S., Castro M.A., Osvaldt A.B., Matte U., Ashton-Prolla P. (2016). miRNA-21 and miRNA-34a Are Potential Minimally Invasive Biomarkers for the Diagnosis of Pancreatic Ductal Adenocarcinoma. Pancreas.

[176] Qu K., Zhang X., Lin T., Liu T., Wang Z., Liu S., Zhou L., Wei J., Chang H., Li K. (2017). Circulating miRNA-21-5p as a diagnostic biomarker for pancreatic cancer: Evidence from comprehensive miRNA expression profiling analysis and clinical validation. Sci. Rep..

[177] Stroese A.J., Ullerich H., Koehler G., Raetzel V., Senninger N., Dhayat S.A. (2018). Circulating microRNA-99 family as liquid biopsy marker in pancreatic adenocarcinoma. J. Cancer Res. Clin. Oncol..

[178] Goto T., Fujiya M., Konishi H., Sasajima J., Fujibayashi S., Hayashi A., Utsumi T., Sato H., Iwama T., Ijiri M. (2018). An elevated expression of serum exosomal microRNA-191, - 21, -451a of pancreatic neoplasm is considered to be efficient diagnostic marker. BMC Cancer.

[179] Abue M., Yokoyama M., Shibuya R., Tamai K., Yamaguchi K., Sato I., Tanaka N., Hamada S., Shimosegawa T., Sugamura K. (2015). Circulating miR-483-3p and miR-21 is highly expressed in plasma of pancreatic cancer. Int. J. Oncol..

[180] Kawamura S., Iinuma H., Wada K., Takahashi K., Minezaki S., Kainuma M., Shibuya M., Miura F., Sano K. (2019). Exosome-encapsulated microRNA-4525, microRNA-451a and microRNA-21 in portal vein blood is a high-sensitive liquid biomarker for the selection of high-risk pancreatic ductal adenocarcinoma patients. J. Hepato-Biliary-Pancreat. Sci..

[181] Mikamori M., Yamada D., Eguchi H., Hasegawa S., Kishimoto T., Tomimaru Y., Asaoka T., Noda T., Wada H., Kawamoto K. (2017). MicroRNA-155 Controls Exosome Synthesis and Promotes Gemcitabine Resistance in Pancreatic Ductal Adenocarcinoma. Sci. Rep..

[182] Mazza T., Gioffreda D., Fontana A., Biagini T., Carella M., Palumbo O., Maiello E., Bazzocchi F., Andriulli A., Tavano F. (2020). Clinical Significance of Circulating miR-1273g-3p and miR-122-5p in Pancreatic Cancer. Front. Oncol..

[183] Marin A.M., Mattar S.B., Amatuzzi R.F., Chammas R., Uno M., Zanette D.L., Aoki M.N. (2022). Plasma Exosome-Derived microRNAs as Potential Diagnostic and Prognostic Biomarkers in Brazilian Pancreatic Cancer Patients. Biomolecules.

[184] Takahasi K., Iinuma H., Wada K., Minezaki S., Kawamura S., Kainuma M., Ikeda Y., Shibuya M., Miura F., Sano K. (2018). Usefulness of exosome-encapsulated microRNA-451a as a minimally invasive biomarker for prediction of recurrence and prognosis in pancreatic ductal adenocarcinoma. J. Hepato-Biliary-Pancreat. Sci..

[185] Michael Traeger M., Rehkaemper J., Ullerich H., Steinestel K., Wardelmann E., Senninger N., Abdallah Dhayat S. (2018). The ambiguous role of microRNA-205 and its clinical potential in pancreatic ductal adenocarcinoma. J. Cancer Res. Clin. Oncol..

[186] Shi W., Wartmann T., Accuffi S., Al-Madhi S., Perrakis A., Kahlert C., Link A., Venerito M., Keitel-Anselmino V., Bruns C. (2024). Integrating a microRNA signature as a liquid biopsy-based tool for the early diagnosis and prediction of potential therapeutic targets in pancreatic cancer. Br. J. Cancer.

[187] Liu J., Zhu C., Zhang L., Lu H., Wang Z., Lv J., Fan C. (2020). MicroRNA-1469-5p promotes the invasion and proliferation of pancreatic cancer cells via direct regulating the NDRG1/NF-κB/E-cadherin axis. Hum. Cell.

[188] Shams R., Saberi S., Zali M., Sadeghi A., Ghafouri-Fard S., Aghdaei H.A. (2020). Identification of potential microRNA panels for pancreatic cancer diagnosis using microarray datasets and bioinformatics methods. Sci. Rep..

[189] Yan Q., Hu D., Li M., Chen Y., Wu X., Ye Q., Wang Z., He L., Zhu J. (2020). The Serum MicroRNA Signatures for Pancreatic Cancer Detection and Operability Evaluation. Front. Bioeng. Biotechnol..

[190] Vietsch E.E., Peran I., Suker M., van den Bosch T.P.P., van der Sijde F., Kros J.M., van Eijck C.H.J., Wellstein A. (2019). Immune-Related Circulating miR-125b-5p and miR-99a-5p Reveal a High Recurrence Risk Group of Pancreatic Cancer Patients after Tumor Resection. Appl. Sci..

[191] Gablo N., Trachtova K., Prochazka V., Hlavsa J., Grolich T., Kiss I., Srovnal J., Rehulkova A., Lovecek M., Skalicky P. (2020). Identification and Validation of Circulating Micrornas as Prognostic Biomarkers in Pancreatic Ductal Adenocarcinoma Patients Undergoing Surgical Resection. J. Clin. Med..

[192] Seyed Salehi A., Parsa-Nikoo N., Roshan-Farzad F., Shams R., Fathi M., Asaszadeh Aghdaei H., Behmanesh A. (2022). MicroRNA-125a-3p, -4530, and -92a as a Potential Circulating MicroRNA Panel for Noninvasive Pancreatic Cancer Diagnosis. Dis. Markers.

[193] Zou X., Wei J., Huang Z., Zhou X., Lu Z., Zhu W., Miao Y. (2019). Identification of a six-miRNA panel in serum benefiting pancreatic cancer diagnosis. Cancer Med..

[194] Liu G., Shao C., Li A., Zhang X., Guo X., Li J. (2020). Diagnostic Value of Plasma miR-181b, miR-196a, and miR-210 Combination in Pancreatic Cancer. Gastroenterol. Res. Pract..

[195] Ganepola G.A., Rutledge J.R., Suman P., Yiengpruksawan A., Chang D.H. (2014). Novel blood-based microRNA biomarker panel for early diagnosis of pancreatic cancer. World J. Gastrointest. Oncol..

[196] Johansen J.S., Calatayud D., Albieri V., Schultz N.A., Dehlendorff C., Werner J., Jensen B.V., Pfeiffer P., Bojesen S.E., Giese N. (2016). The potential diagnostic value of serum microRNA signature in patients with pancreatic cancer. Int. J. Cancer.

[197] Schultz N.A., Dehlendorff C., Jensen B.V., Bjerregaard J.K., Nielsen K.R., Bojesen S.E., Calatayud D., Nielsen S.E., Yilmaz M., Holländer N.H. (2014). MicroRNA biomarkers in whole blood for detection of pancreatic cancer. JAMA.

[198] Nakamura K., Zhu Z., Roy S., Jun E., Han H., Munoz R.M., Nishiwada S., Sharma G., Cridebring D., Zenhausern F. (2022). An Exosome-based Transcriptomic Signature for Noninvasive, Early Detection of Patients With Pancreatic Ductal Adenocarcinoma: A Multicenter Cohort Study. Gastroenterology.

[199] Masterson A.N., Chowdhury N.N., Fang Y., Yip-Schneider M.T., Hati S., Gupta P., Cao S., Wu H., Schmidt C.M., Fishel M.L. (2023). Amplification-Free, High-Throughput Nanoplasmonic Quantification of Circulating MicroRNAs in Unprocessed Plasma Microsamples for Earlier Pancreatic Cancer Detection. ACS Sens..

[200] Wei J., Yang L., Wu Y.N., Xu J. (2020). Serum miR-1290 and miR-1246 as Potential Diagnostic Biomarkers of Human Pancreatic Cancer. J. Cancer.

[201] Kandimalla R., Shimura T., Mallik S., Sonohara F., Tsai S., Evans D.B., Kim S.C., Baba H., Kodera Y., Von Hoff D. (2022). Identification of Serum miRNA Signature and Establishment of a Nomogram for Risk Stratification in Patients With Pancreatic Ductal Adenocarcinoma. Ann. Surg..

[202] Nishiwada S., Cui Y., Sho M., Jun E., Akahori T., Nakamura K., Sonohara F., Yamada S., Fujii T., Han I.W. (2022). Transcriptomic Profiling Identifies an Exosomal microRNA Signature for Predicting Recurrence Following Surgery in Patients With Pancreatic Ductal Adenocarcinoma. Ann. Surg..

[203] Álvarez-Hilario L.G., Salmerón-Bárcenas E.G., Ávila-López P.A., Hernández-Montes G., Aréchaga-Ocampo E., Herrera-Goepfert R., Albores-Saavedra J., Manzano-Robleda M.D.C., Saldívar-Cerón H.I., Martínez-Frías S.P. (2023). Circulating miRNAs as Noninvasive Biomarkers for PDAC Diagnosis and Prognosis in Mexico. Int. J. Mol. Sci..

[204] Li W., Gonzalez-Gonzalez M., Sanz-Criado L., Garcia-Carbonero N., Celdran A., Villarejo-Campos P., Minguez P., Pazo-Cid R., Garcia-Jimenez C., Orta-Ruiz A. (2022). A Novel PiRNA Enhances CA19-9 Sensitivity for Pancreatic Cancer Identification by Liquid Biopsy. J. Clin. Med..

[205] Saha B., Chakravarty S., Ray S., Saha H., Das K., Ghosh I., Mallick B., Biswas N.K., Goswami S. (2024). Correlating tissue and plasma-specific piRNA changes to predict their possible role in pancreatic malignancy and chronic inflammation. Biomed. Rep..

[206] Jin F., Yang L., Wang W., Yuan N., Zhan S., Yang P., Chen X., Ma T., Wang Y. (2021). A novel class of tsRNA signatures as biomarkers for diagnosis and prognosis of pancreatic cancer. Mol. Cancer.

[207] Liu Y., Feng W., Liu W., Kong X., Li L., He J., Wang D., Zhang M., Zhou G., Xu W. (2019). Circulating lncRNA ABHD11-AS1 serves as a biomarker for early pancreatic cancer diagnosis. J. Cancer.

[208] Liu P., Sun Q.Q., Liu T.X., Lu K., Zhang N., Zhu Y., Chen M. (2019). Serum lncRNA-UFC1 as a potential biomarker for diagnosis and prognosis of pancreatic cancer. Int. J. Clin. Exp. Pathol..

[209] Guo Z., Wang X., Yang Y., Chen W., Zhang K., Teng B., Huang C., Zhao Q., Qiu Z. (2020). Hypoxic Tumor-Derived Exosomal Long Noncoding RNA UCA1 Promotes Angiogenesis via miR-96-5p/AMOTL2 in Pancreatic Cancer. Mol. Ther. Nucleic Acids.

[210] Li Z., Jiang P., Li J., Peng M., Zhao X., Zhang X., Chen K., Zhang Y., Liu H., Gan L. (2018). Tumor-derived exosomal lnc-Sox2ot promotes EMT and stemness by acting as a ceRNA in pancreatic ductal adenocarcinoma. Oncogene.

[211] Takahashi K., Ota Y., Kogure T., Suzuki Y., Iwamoto H., Yamakita K., Kitano Y., Fujii S., Haneda M., Patel T. (2020). Circulating extracellular vesicle-encapsulated HULC is a potential biomarker for human pancreatic cancer. Cancer Sci..

[212] Ou Z.L., Luo Z., Lu Y.B. (2019). Long non-coding RNA HULC as a diagnostic and prognostic marker of pancreatic cancer. World J. Gastroenterol..

[213] Wang Y., Li Z., Zheng S., Zhou Y., Zhao L., Ye H., Zhao X., Gao W., Fu Z., Zhou Q. (2015). Expression profile of long non-coding RNAs in pancreatic cancer and their clinical significance as biomarkers. Oncotarget.

[214] Guo X.B., Yin H.S., Wang J.Y. (2018). Evaluating the diagnostic and prognostic value of long non-coding RNA SNHG15 in pancreatic ductal adenocarcinoma. Eur. Rev. Med. Pharmacol. Sci..

[215] Ma Y., Hu M., Zhou L., Ling S., Li Y., Kong B., Huang P. (2019). Long non-coding RNA HOTAIR promotes cancer cell energy metabolism in pancreatic adenocarcinoma by upregulating hexokinase-2. Oncol. Lett..

[216] Ge J.N., Yan D., Ge C.L., Wei M.J. (2020). LncRNA C9orf139 can regulate the growth of pancreatic cancer by mediating the miR-663a/Sox12 axis. World J. Gastrointest. Oncol..

[217] Du W., Lei C., Wang Y., Ding Y., Tian P. (2021). LINC01232 Sponges Multiple miRNAs and Its Clinical Significance in Pancreatic Adenocarcinoma Diagnosis and Prognosis. Technol. Cancer Res. Treat..

[218] Hong L., Xu L., Jin L., Xu K., Tang W., Zhu Y., Qiu X., Wang J. (2022). Exosomal circular RNA hsa_circ_0006220, and hsa_circ_0001666 as biomarkers in the diagnosis of pancreatic cancer. J. Clin. Lab. Anal..

[219] Yang F., Liu D.Y., Guo J.T., Ge N., Zhu P., Liu X., Wang S., Wang G.X., Sun S.Y. (2017). Circular RNA circ-LDLRAD3 as a biomarker in diagnosis of pancreatic cancer. World J. Gastroenterol..

[220] Xu K., Qiu Z., Xu L., Qiu X., Hong L., Wang J. (2021). Increased levels of circulating circular RNA (hsa_circ_0013587) may serve as a novel biomarker for pancreatic cancer. Biomark. Med..

[221] Seimiya T., Otsuka M., Iwata T., Tanaka E., Sekiba K., Shibata C., Moriyama M., Nakagawa R., Maruyama R., Koike K. (2021). Aberrant expression of a novel circular RNA in pancreatic cancer. J. Hum. Genet..

[222] Xu C., Jun E., Okugawa Y., Toiyama Y., Borazanci E., Bolton J., Taketomi A., Kim S.C., Shang D., Von Hoff D. (2024). A Circulating Panel of circRNA Biomarkers for the Noninvasive and Early Detection of Pancreatic Ductal Adenocarcinoma. Gastroenterology.

[223] Li Z., Yanfang W., Li J., Jiang P., Peng T., Chen K., Zhao X., Zhang Y., Zhen P., Zhu J. (2018). Tumor-released exosomal circular RNA PDE8A promotes invasive growth via the miR-338/MACC1/MET pathway in pancreatic cancer. Cancer Lett..

[224] Li J., Li Z., Jiang P., Peng M., Zhang X., Chen K., Liu H., Bi H., Liu X., Li X. (2018). Circular RNA IARS (circ-IARS) secreted by pancreatic cancer cells and located within exosomes regulates endothelial monolayer permeability to promote tumor metastasis. J. Exp. Clin. Cancer Res..

[225] Zeng Z., Zhao Y., Chen Q., Zhu S., Niu Y., Ye Z., Hu P., Chen D., Xu P., Chen J. (2021). Hypoxic exosomal HIF-1α-stabilizing circZNF91 promotes chemoresistance of normoxic pancreatic cancer cells via enhancing glycolysis. Oncogene.

[226] Liu X., Zhong L., Jiang W., Wen D. (2021). Repression of circRNA_000684 inhibits malignant phenotypes of pancreatic ductal adenocarcinoma cells via miR-145-mediated KLF5. Pancreatology.

[227] Shen X., Chen Y., Li J., Huang H., Liu C., Zhou N. (2021). Identification of Circ_001569 as a Potential Biomarker in the Diagnosis and Prognosis of Pancreatic Cancer. Technol. Cancer Res. Treat..

[228] Lin J., Wang X., Zhai S., Shi M., Peng C., Deng X., Fu D., Wang J., Shen B. (2022). Hypoxia-induced exosomal circPDK1 promotes pancreatic cancer glycolysis via c-myc activation by modulating miR-628-3p/BPTF axis and degrading BIN1. J. Hematol. Oncol..

[229] Abdullah S.T., Abdullah S.R., Hussen B.M., Younis Y.M., Rasul M.F., Taheri M. (2023). Role of circular RNAs and gut microbiome in gastrointestinal cancers and therapeutic targets. Noncoding RNA Res..

